# RNAdualPF: software to compute the dual partition function with sample applications in molecular evolution theory

**DOI:** 10.1186/s12859-016-1280-6

**Published:** 2016-10-19

**Authors:** Juan Antonio Garcia-Martin, Amir H. Bayegan, Ivan Dotu, Peter Clote

**Affiliations:** 1 Biology Department, Boston College, 140 Commonwealth Avenue, Chestnut Hill, 02467 MA USA; 2 Research Programme on Biomedical Informatics (GRIB), Department of Experimental and Health Sciences, Universitat Pompeu Fabra, IMIM (Hospital del Mar Medical Research Institute), Dr. Aiguader, 88, Barcelona, Spain; 3 Present Address: Systems Biology Program Centro Nacional de Biotecnología Consejo Superior de Investigaciones Científicas (CSIC) C/ Darwin 3, Madrid, 28049 Spain

**Keywords:** RNA secondary structure, Partition function, Boltzmann ensemble, Robustness

## Abstract

**Background:**

RNA inverse folding is the problem of finding one or more sequences that fold into a user-specified target structure *s*_0_, i.e. whose minimum free energy secondary structure is identical to the target *s*_0_. Here we consider the ensemble of all RNA sequences that have low free energy with respect to a given target *s*_0_.

**Results:**

We introduce the program RNAdualPF, which computes the *dual partition function* *Z* ^∗^, defined as the sum of Boltzmann factors exp(−*E*(**a,s**_0_)/*RT*) of all RNA nucleotide sequences **a** compatible with target structure *s*_0_. Using RNAdualPF, we efficiently sample RNA sequences that approximately fold into *s*_0_, where additionally the user can specify IUPAC sequence constraints at certain positions, and whether to include dangles (energy terms for stacked, single-stranded nucleotides). Moreover, since we also compute the *dual partition function* *Z* ^∗^(*k*) over all sequences having GC-content *k*, the user can require that all sampled sequences have a precise, specified GC-content.

Using *Z* ^∗^, we compute the *dual expected energy* 〈*E* ^∗^〉, and use it to show that natural RNAs from the Rfam 12.0 database have *higher* minimum free energy than expected, thus suggesting that functional RNAs are under evolutionary pressure to be only marginally thermodynamically stable.

We show that *C. elegans* precursor microRNA (pre-miRNA) is significantly *non-robust* with respect to mutations, by comparing the robustness of each wild type pre-miRNA sequence with 2000 [resp. 500] sequences of the same GC-content generated by RNAdualPF, which approximately [resp. exactly] fold into the wild type target structure. We confirm and strengthen earlier findings that precursor microRNAs and bacterial small noncoding RNAs display plasticity, a measure of structural diversity.

**Conclusion:**

We describe RNAdualPF, which rapidly computes the *dual partition function* *Z* ^∗^ and samples sequences having low energy with respect to a target structure, allowing sequence constraints and specified GC-content. Using different inverse folding software, another group had earlier shown that pre-miRNA is mutationally robust, even controlling for compositional bias. Our opposite conclusion suggests a cautionary note that computationally based insights into molecular evolution may heavily depend on the software used.

C/C++-software for RNAdualPF is available at http://bioinformatics.bc.edu/clotelab/RNAdualPF.

**Electronic supplementary material:**

The online version of this article (doi:10.1186/s12859-016-1280-6) contains supplementary material, which is available to authorized users.

## Background

In [1], Borenstein and Ruppin define *neutrality* of an RNA sequence **a**=*a*_1_,…,*a*_*n*_ by $\eta (\mathbf {a}) = 1-\frac {\langle d \rangle }{n}$, where in this section 〈*d*〉 denotes the average, taken over all 3*n* single-point mutants of **a**, of the base pair distance *d*_BP_ between the minimum free energy (MFE) structure *s*_0_ of **a** and the MFE structures of single-point mutants of **a**. An RNA sequence **a** is then defined to be *robust* if *η*(**a**) is greater than the average neutrality of 1000 control sequences generated by the program RNAinverse [2], which fold into the same target structure *s*_0_. The main finding of [1] is that precursor microRNAs (pre-miRNA) exhibit a significantly higher level of mutational robustness than random RNA sequences having the same structure. To control for sequence composition bias in their computational study, the authors selected sequences from the output of RNAinverse, whose dinucleotide composition was similar to that of wild type pre-miRNA (Jensen-Shannon divergence less than 0.01). Since the filtering step required enormous run time and computational resources, the authors restricted their attention to a small set of 211 microRNAs, generating only 100 control sequences per microRNA. Borenstein and Ruppin conclude that robustness of precursor microRNAs is not the byproduct of a base composition bias or of thermodynamic stability.

Subsequently Rodrigo et al. [3] undertook a similar analysis for bacterial small RNAs, also using the program RNAinverse, albeit using somewhat different definitions – precise definitions are given in “Formal definitions of robustness” section. The main finding of [3] was that bacterial sncRNAs are not significantly robust when compared with 1000 sequences having the same structure, as computed by RNAinverse; however, bacterial sncRNAs tend to be significantly *plastic*, in the sense that the ensemble of low energy structures is structurally diverse. Unlike the case of precursor microRNAs [1], Rodrigo et al. did not control for sequence compositional bias.

This raises the question of whether the control sequences analyzed in [1, 3] are *representative* or to what extent features shared by sequences output by the program RNAinverse are artifacts of the program used. Indeed, the number of RNA sequences that fold into a given target structure can be astronomically large. Over a few weeks, before we elected to terminate the execution, our state-of-the-art inverse folding software RNAiFold [4] generated 273,926,421 many 52-nt sequences that fold exactly into the MFE secondary structure *s*_0_ of HIV-1 ribosomal frameshift stimulating signal from the Gag-Pol overlap region AF033819.3/1631-1682, and which additionally code 17-mer peptides in the Gag and Pol reading frames having amino acids that appear in Gag/Pol peptides found in the Los Alamos HIV-1 database [5]. The number of 52 nt RNA sequences that fold into target *s*_0_ without additionally imposing the constraint of coding particular peptides in overlapping Gag/Pol reading frames is certain to dwarf the previous number. Moreover, the number of sequences that fold into the MFE structure of an animal precursor microRNA (length 68 to 91 nt [6]) or into the MFE structure of bacterial sncRNA (length 53-436 nt [3]) is certain to be even more daunting.

Different inverse folding algorithms have adopted different strategies to generate sequences that fold into a user-specified target secondary structure *s*_0_. For instance, RNAinverse [2, 7] performs an *adaptive walk*, in one step of which a nucleotide in the current sequence is mutated and subsequently accepted if the base pair distance between the minimum free energy (MFE) structure of the mutated sequence and the target structure *s*_0_ is reduced. NUPACK Design [8] selects a candidate mutation position with probability proportional to its contribution to the *ensemble defect* (Boltzmann-weighted Hamming distance to the vector representation of *s*_0_, where *s*_0_[*i*]=*j* indicates (*i,j*)∈*s*_0_ and *s*_0_[*i*]=*i* indicates *i* is unpaired in *s*_0_). RNAiFold CP-design [4, 9] uses constraint programming to systematically explore the search tree of all inverse folding solutions in an order determined by certain heuristics. Accordingly, one cannot claim that the collection of sequences generated by any particular inverse folding algorithm is *representative* of the astronomically large space of all inverse folding solutions – indeed, each inverse folding algorithm has an inherent but *unknown* bias.

In this paper, we describe the algorithm RNAdualPF, which generates sequences which have low free energy with respect to a user-specified target structure *s*_0_ – i.e. the inherent bias of RNAdualPF is known, unlike the situation of other inverse folding algorithms. We show that RNAdualPF is extremely fast software for generating sequences that *approximately* fold into *s*_0_; moreover, in a postprocessing step, one can filter the output of RNAdualPF to select sequences that exactly fold into *s*_0_. RNAdualPF additionally allows the user to specify IUPAC codes to constrain certain nucleotide positions as well as to control the GC-content of all generated sequences. Sampling is performed in a manner distinct but somewhat analogous to that by which Sfold [10] and RNAsubopt -p [2] sample representative secondary structures from the Boltzmann ensemble of all structures of a given sequence. Using RNAdualPF, we perform a pilot study that is similar, though not identical, to that of [1, 3] for two classes of RNA: 250 *C. elegans* precursor microRNA from miRBase [11] and the bacterial small noncoding RNAs previously analyzed in [3].

Finally, it should be noted that, although RNAdualPF was developed entirely independently of the work of Reinharz et al. [12], one can view our C-program as an extension of Python program IncaRNAtion [12] to the full Turner energy model, where additionally GC-content is rigorously handled. This point will be discussed further in the Conclusion.

### Formal definitions of robustness

Let **a**=*a*_1_,…,*a*_*n*_ denote an arbitrary RNA sequence, where $a_{i} \in \mathcal {N} = \{\text {A,\,U,\,G,\,C}\}$, a secondary structure *s* of **a** is a set of base pairs (*i,j*) satisfying the following conditions: (1) If (*i,j*)∈*s* then *a*_*i*_,*a*_*j*_ constitute a Watson-Crick or GU wobble pair, i.e. $ij \in \mathcal {B}$ which is the set {AU,UA,GC,CG,GU,UG}. (2) If (*i,j*)∈*s* then *i*+*θ*<*j*, where *θ*=3 (a minimum assumed for steric hindrance). (3) If (*i,j*)∈*s* and (*k*,*ℓ*)∈*s*, then either *i*<*k*<*ℓ*<*j* or *k*<*i*<*j*<*ℓ* or *i*<*j*<*k*<*ℓ* or *k*<*ℓ*<*i*<*j*. The collection of all secondary structures of the RNA sequence **a** is denoted ${\mathbb {S}\mathbb {S}}(\mathbf {a})$, and the free energy [13] of *s* is denoted by *E*(**a,s**), or simply by *E*(*s*) provided that the sequence **a** is clear from context. The *Boltzmann probability* *p*(*s*)=*p*_**a**_(*s*) for structure *s* of **a** is defined by exp(−*E*(**a,s**)/*RT*)/*Z*, where the partition function $Z = Z(\mathbf {a}) = \sum _{s \in {\mathbb {S}\mathbb {S}}(\mathbf {a})} \exp (-E(\mathbf {a},s)/RT)$. Given two secondary structures *s,t* of **a**, the *base pair distance* *d*_BP_(*s,t*) between *s* and *t* is defined to be the size of the symmetric difference of *s,t*, i.e. |*s*−*t*|+|*t*−*s*|.

In [3], Rodrigo et al. define *intrinsic distance*

1 $$\begin{array}{*{20}l}  d_{0}(\mathbf{a}) =\sum_{s,t} p(s) \cdot p(t) \cdot d_{\text{\textsc{bp}}}(s,t) \end{array} $$

i.e. intrinsic distance is another name for *ensemble diversity* earlier defined in [14], and computed by Vienna RNA Package [2]. *Plasticity* is defined in [3] to be *normalized ensemble diversity*; i.e.

2 $$\begin{array}{*{20}l}  P(\mathbf{a}) = \frac{d_{0}(\mathbf{a})}{n/2} \end{array} $$

obtained by dividing ensemble diversity by (essentially) the maximum possible number *n*/2 of base pairs in a structure of **a**. Given two RNA sequences **a**=*a*_1_,…,*a*_*n*_ and **b**=*b*_1_,…,*b*_*n*_ of the same length *n*, Rodrigo et al. define *d*_1_(**a**,**b**) to be the expected base pair distance between structures of **a** and structures of **b** minus the ensemble diversity of **a**, i.e.

3 $$\begin{array}{*{20}l}  d_{1}(\mathbf{a},\mathbf{b}) &= \sum_{s \in {\mathbb{SS}}(\mathbf{a})} \sum_{t \in {\mathbb{SS}}(\mathbf{b})} p_{\mathbf{a}}(s) \cdot p_{\mathbf{b}}(t) \cdot d_{\text{\sc bp}}(s,t) - d_{0}(\mathbf{a}) \end{array} $$

Since *d*_1_ is not symmetric, this measure is not a metric. In contrast, *ensemble distance* as described in [14] is a valid metric, defined by the following:

4 $$ \begin{aligned} D_{\text{\textsc{v}}}(\mathbf{a},\mathbf{b}) &=\sqrt{\sum_{s \in {\mathbb{S}\mathbb{S}}(\mathbf{a})} \sum_{t \in {\mathbb{S}\mathbb{S}}(\mathbf{b})}p_{\mathbf{a}}(s) \cdot p_{\mathbf{b}}(t) \cdot d_{\text{\textsc{bp}}}(s,t) - \frac{d_{0}(\mathbf{a}) + d_{0}(\mathbf{b})}{2}}\\ &=\sqrt{\sum_{i<j} (p_{i,j}(a) - p_{i,j}(b))^{2} } \end{aligned}  $$

In [3], Rodrigo et al. define the *mutational robustness*

5 $$\begin{array}{*{20}l}  R_{m}(\mathbf{a}) &=&1 -\frac{\langle d_{1}(\mathbf{a},\mathbf{a'}) \rangle}{n/2} \end{array} $$

where 〈*d*_1_(**a**,**a’**)〉 denotes the average value of *d*_1_(**a**,**a’**) taken over all single point mutants **a’** of **a**. Since *d*_1_(**a**,**a’**) is not a true metric, we replace it by the metric *D*_V_(**a**,**b**) in our computation of mutational robustness. Clearly both notions are closely related.

## Implementation

In [15], McCaskill described a cubic time algorithm to compute the *partition function*

6 $$\begin{array}{*{20}l}  Z &= Z(\mathbf{a}) = \sum_{s \in \mathbb{S}\mathbb{S}(\mathbf{a})} \exp(-E(\mathbf{a},s)/RT) \end{array} $$

for an RNA sequence **a**=*a*_1_,…,*a*_*n*_, where the sum is taken over all secondary structures $\mathbb {S}\mathbb {S}(\mathbf {a})$ of **a**, *E*(**a,s**) denotes the free energy for the structure *s* of **a** with respect to the Turner energy parameters [13], *R* denotes the universal gas constant and *T* is absolute temperature. Subsequently Ding and Lawrence [16] described how to use the partition function together with a simple backtracking strategy to *sample* secondary structures of **a** from the Boltzmann ensemble of low energy structures.

If *s*_0_ is a given secondary structure of length *n*, we define the *dual partition function*

7 $$\begin{array}{*{20}l}  Z^{*} &= Z^{*}(s_{0}) = \sum_{\mathbf{a} \in \mathbb{A}\mathbb{A}(s_{0})} \exp(-E(\mathbf{a},s_{0})/RT) \end{array} $$

where the sum is taken over all RNA sequences **a**=*a*_1_,…,*a*_*n*_ of length *n* that are compatible with structure *s*_0_, i.e. *a*_*i*_,*a*_*j*_ constitute a Watson-Crick or wobble pair for each base pair (*i,j*)∈*s*_0_. The set of all RNA sequences that are compatible with *s*_0_ is denoted by $\mathbb {AA}(s_{0})$. Note that if a sequence **a** is not compatible with the target structure *s*_0_, then the energy *E*(**a,s**_0_) is infinite, so the corresponding Boltzmann factor exp(−*E*(**a,s**_0_)/*RT*) is zero and the sum in Eq. (7) could have been written over all sequences of the same length as *s*_0_. Here we describe the efficient software RNAdualPF to compute the *dual partition function* *Z* ^∗^ and to sample from the low energy ensemble of *sequences* that are compatible with a given secondary structure *s*_0_.

### Dual partition function

If *s* is a secondary structure on sequence **a**=*a*_1_,…,*a*_*n*_, then the *length* of *s*, denoted by *ℓ*(*s*), is equal to *n*, while the *size* of *s*, denoted by |*s*|, is the number of base pairs belonging to *s*. Similarly, if secondary structure *s* is restricted to the interval [*i,j*], where 1≤*i*≤*j*≤*n*, then the length of the restriction of *s* to [*i,j*], denoted by *ℓ*(*s*[*i,j*]), is equal to *j*−*i*+1, while the size of the restriction of *s* to [*i,j*], denoted by |*s*[*i,j*]|, is the number of base pairs (*X,Y*) of *s* that satisfy *i*≤*x*<*y*≤*j*.

Given an RNA sequence **a**=*a*_1_,…,*a*_*n*_, the McCaskill algorithm [15] computes the partition function *Z*(**a**) defined in Eq. (6). When **a** is clear from context, *Z*(**a**) is usually denoted by *Z*.

Given a target secondary structure *s*_0_, we describe below an algorithm to compute the *dual partition function* *Z* ^∗^(*s*_0_), defined as the sum of all Boltzmann factors exp(−*E*(**a,s**_0_)), where the sum is taken over all RNA sequences $\mathbf {a} \in \mathbb {A}\mathbb {A}(s_{0})$. Unlike the McCaskill algorithm, which requires time that is cubic in the length of **a**, the algorithm presented below requires time that is (essentially) linear^1^ in the length of *s*_0_. Our algorithm is motivated by the initialization step of the algorithm INFO-RNA [17], in which a sequence is determined, for which the free energy with respect to target structure *s*_0_ is a minimum – i.e. INFO-RNA determines argmin_**a**_ *E*(**a,s**_0_).

The algorithm specification requires the notation *Z* ^∗^(*i,j*;*X,Y*), which denotes the sum

8 $$\begin{array}{*{20}l}  Z^{*}(i,j;x,y) = \sum\limits_{\mathbf{a}[i,j],a_{i}=x,a_{j}=y} \exp\left(-E\left(\mathbf{a}[i,j],s_{0}[i,j]\right)/RT\right) \end{array} $$

of Boltzmann factors for sequences **a**[*i,j*]=*a*_*i*_,…,*a*_*j*_ for which *a*_*i*_=*x,a*_*j*_=*y*, and for the restriction *s*_0_[*i,j*], defined by

9 $$\begin{array}{*{20}l}  s_{0}[i,j] = \left\{ (x,y) \in s_{0}: i \leq x < y \leq j\right\}. \end{array} $$

The function *Z* ^∗^(*i,j*;*X,Y*) is defined for all base pairs (*i,j*)∈*s*_0_; these values will be stored in an array, whose rows index base pairs of *s*_0_, and whose columns are indexed by the six canonical base pairs GC, CG, AU, UA, GU, UG (see example in Table 1). Once *Z* ^∗^(*i,j*;*X,Y*) has been computed for all base pairs that are *visible*, i.e. for which there is no base pair (*X,Y*) for which *x*<*i*<*j*<*y*, we can compute the full partition function *Z* ^∗^(*s*_0_).

**Table 1:** Base pair dual partition function table. Given the target structure with sequence constraints depicted in Fig. 2, RNAdualPF computes and stores all the partial *dual partition function* values for the substructures enclosed by each base pair

Index	i	j	Type	*AU*	*CG*	*GC*	*UA*	*GU*	*UG*	*Z* ^∗^(*i,j*)
1	18	23	Tetraloop	0.000	0.000	0.364	0.000	0.000	0.000	0.364
2	17	24	Stack	10.977	17.859	76.923	10.977	10.977	3.525	131.238
3	16	26	R. bulge	11.690	70.834	184.603	12.771	13.347	3.915	297.160
4	6	10	Triloop	0.004	0.010	0.010	0.004	0.004	0.004	0.038
5	5	11	Stack	0.750	3.022	5.234	0.899	0.960	0.256	11.120
6	3	13	Int. loop	109.842	256.875	424.976	108.653	117.851	108.132	1126.330
7	2	14	Stack	10853.104	86208.448	170643.321	12575.544	13285.398	3647.077	297212.891
8	1	27	Multiloop	1558.575	7895.583	7895.583	1558.575	1558.575	1558.575	22025.464
9	1	28	*S*_0_	–	–	–	–	–	–	88101.856

The first column indicates the *base pair index* which dictates the order in which the dual partition function is computed for different loops closed by the base pair (i,j), where we the *index* of base pair (*i,j*) is defined to be the rank of (*i,j*) in the total ordering defined in Eq. (10). Columns *i* and *j* indicate the opening and closing positions of each base pair. Type indicates the type of element in the secondary structure closed by each base pair, where R. bulge stands for right bulge, Stack for stacking base pair, and Int. loop for interior loop. The *dual partition function* *Z* ^∗^(*i,j*) of the substructure closed by base pair (*i,j*) appears in the rightmost column, while the partition function *Z* ^∗^(*i,j,X,Y*) for each of the six canonical base pairs is given in columns 5-10. Note that for base pair 1, sequence constraints depicted in Fig. 2 force *i* and *j* to be instantiated respectively to G and C, hence the dual partition function *Z* ^∗^(*i,j*;*X,Y*) is zero for any base pair different than GC. The last column of the last row of the table shows the total dual partition function *Z* ^∗^(*s*_0_) for the target structure *s*_0_

Following [17], we define a total ordering on base pairs (*i,j*) belonging to the target structure *s*_0_ that satisfy the following precedence rule for any two base pairs (*i,j*),(*X,Y*).

10 $$\begin{array}{*{20}l}  (i,j) &\prec (x,y) \Leftrightarrow x<i<j<y~ \text{or}~ i < j < x < y \end{array} $$

From this ordering, we assign a *base pair index* to each base pair (*i,j*), which is defined to be the rank of (*i,j*) in the total ordering.

The following definitions correspond to the Turner nearest neighbor energy model [13], which is an additive loop model where a loop closed by external base pair (*i,j*) is designated as a *k*-loop, if the loop contains *k* base pairs interior to (*i,j*). Therefore, hairpin loops are 0-loops; base pair stacks, bulge loops and internal loops are 1-loops; and multiloops are *k*-loops for *k*≥2 (also called (*k*+1)-way junctions), where the additional count is due to the outer component adjacent to (*i,j*) [18].

Since AU-base pairs that close a loop are energetically unfavorable, in the Turner energy model, there is an AU-penalty we now define:

11 $$\begin{array}{@{}rcl@{}} e_{AU}(i,j,X,Y) &= \left\{\begin{array}{rl} 0.5 &\text{if~} (i,j) \text{~is the outermost pair in a stem of~} s_{0}, \text{~having AU,UA,GU,UG}\\ 0 &\text{otherwise.} \end{array} \right. \end{array} $$

This AU-penalty is applied only if (*i,j*) is a base pair adjacent to a triloop, a bulge, an internal loop or a multiloop, or if it is the outermost base pair of an external loop in target structure *s*_0_, and (*i,j*) is instantiated by one of the pairs AU, UA, GU, UG. When base-paired positions *i,j* are clear from the context, we write *e*_*AU*_(*X,Y*).

Here, we assume that in parsing the input target structure, a list *BPcloseELorML* has been created of those base pairs (*i,j*), which close either an external loop or a multiloop. Let *I* be the indicator function, it follows that if (*i,j*) closes an external loop or multiloop, then $\exp \left (-\frac {I[(i,j) \in BPcloseELorML] \cdot e_{AU}(X,Y)}{RT} \right)$ is the Boltzmann factor for a special AU-penalty, otherwise this factor equals 1. For clarity in the notation, this factor is denoted by $e^{(-\frac {e^{I}_{AU}(X,Y)}{RT})}$. Note that this term is distinct from the factor $\exp (-\frac {e_{AU}(X,Y)}{RT})$ applied to base pairs adjacent to a triloop, a bulge or an internal loop, which does not depend on the indicator function.

#### Hairpins

Let (*i,j*) close a hairpin in *s*_0_. The hairpin free energy term *H*(*j*−*i*−1), arising solely from entropic considerations, is defined by

12 $$ \begin{aligned} &H(j-i-1)\\ &\quad= \left\{ \begin{array}{ll} hairpinE(j-i-1) &\text{if~} j-i-1 \leq 30\\ hairpinE(30)+1.75 RT \ln\left(\frac{j-i-1}{30} \right) &\text{otherwise}\\ \end{array} \right. \end{aligned}  $$

where *hairpinE*(*j*−*i*−1) designates the hairpin free energy obtained from table look-up, when *j*−*i*−1≤30.

**Triloop**

Let *TriLoop*_*X,Y*_ denote the collection of special triloops, *xabcy*, having an energy bonus *triloopE*(*xabcy*).

13 $$ \begin{aligned} Z^{*}(i,j;x,y) &= e^{\left(-\frac{e^{I}_{AU}(x,y)}{RT}\right)} \cdot \exp\left(- \frac{H(j-i-1) + e_{AU}(xy)}{RT}\right) \\ &\quad\times\left(\left(4^{3} - |TriLoop_{x,y}|\right) + \sum_{abc \in TriLoop_{x,y}}\right.\\ &\qquad\quad\exp\left.\left(- \frac{triloopE(xabcy)}{RT}\right) {\vphantom{\sum_{abc \in TriLoop_{x,y}}}}\right) \end{aligned}  $$

**Tetraloop**

Let *TetraLoop*_*X,Y*_ denote the collection of special tetraloops, *xabcdy*, having an energy bonus *tetraloopE*(*xabcdy*). Similarly, given nucleotides $n_{1},n_{2} \in \mathcal {N}$, *TetraLoop*_*X,Y*_(*n*_1_,*n*_2_) denotes the collection of special tetraloops of the form *xn*_1_ *abn*_2_ *y*. Define *Z* ^∗^(*i,j*;*X,Y*) by

14 $$ \begin{aligned} Z^{*}(i,j;x,y) &= e^{\left(-\frac{e^{I}_{AU}(x,y)}{RT}\right)} \cdot \exp\left(- \frac{H(j-i-1)}{RT} \right) \\ & \quad\times\sum_{n_{1},n_{2} \in \mathcal{N}} \left({\vphantom{\quad\times\left\{ \left(4^{2} - |TetraLoop_{x,y}(n_{1},n_{2})|\right) +\sum_{ab \in TetraLoop_{x,y}(n_{1},n_{2})}\right.}}\exp\left(- \frac{mismatch(x,y,n_{1},n_{2})}{RT}\right)\right. \\ &\quad\times\left\{{\vphantom{\sum_{ab \in TetraLoop_{x,y}(n_{1},n_{2})}}} \left(4^{2} - |TetraLoop_{x,y}(n_{1},n_{2})|\right) +\sum_{ab \in TetraLoop_{x,y}(n_{1},n_{2})}\right.\\ &\qquad\left.\left. \exp\left(- \frac{tetraloopE(xn_{1}abn_{2}y)}{RT}\right) {\vphantom{\sum_{ab \in TetraLoop_{x,y}(n_{1},n_{2})}}}\right\}\right) \end{aligned}  $$

**Hexaloop** Let *HexaLoop*_*X,Y*_ denote the collection of special hexaloops, *xabcdefy*, having an energy bonus *hexaloopE*(*xabcdefy*). Similarly, given nucleotides *n*_1_,*n*_2_, *HexaLoop*_*X,Y*_(*n*_1_,*n*_2_) denotes the collection of special hexaloops of the form *xn*_1_ *abcdn*_2_ *y*. Define *Z* ^∗^(*i,j*;*X,Y*) by

15 $$ \begin{aligned} Z^{*}(i,j;x,y) &= e^{\left(-\frac{e^{I}_{AU}(x,y)}{RT}\right)} \cdot \exp\left(- \frac{H(j-i-1)}{RT}\right) \\ &\quad \times\sum_{n_{1},n_{2} \in \mathcal{N}}\left({\vphantom{\exp\left(- \frac{HexaloopE\left(xn_{1}abcdn_{2}y\right)}{RT}{\vphantom{\sum_{ab \in HexaLoop_{x,y}\left(n_{1},n_{2}\right)}}}\right) }}\exp\left(- \frac{mismatch\left(x,y,n_{1},n_{2}\right)}{RT}\right) \right. \\ &\quad\times\left\{{\vphantom{\sum_{ab \in HexaLoop_{x,y}\left(n_{1},n_{2}\right)}}}\left(4^{4} - |HexaLoop_{x,y}\left(n_{1},n_{2}\right)|\right) + \sum_{ab \in HexaLoop_{x,y}\left(n_{1},n_{2}\right)}\right. \\ &\qquad \left. \left. \exp\left(- \frac{HexaloopE\left(xn_{1}abcdn_{2}y\right)}{RT}{\vphantom{\sum_{ab \in HexaLoop_{x,y}\left(n_{1},n_{2}\right)}}}\right) \right\} \right) \end{aligned}  $$

**Hairpin size exceeds four and is different than six**

Define *Z* ^∗^(*i,j*;*X,Y*) by

16 $$ \begin{aligned} Z^{*}(i,j;x,y) &= e^{\left(-\frac{e^{I}_{AU}(x,y)}{RT}\right)} \cdot \exp\left(- \frac{H(j-i-1)}{RT} \right) \\ & \quad\times\left(\sum_{n_{1},n_{2} \in \mathcal{N}} \exp\left(- \frac{mismatch\left(x,y,n_{1},n_{2}\right)}{RT} \right) \cdot 4^{j-i-3} \right) \end{aligned}  $$

#### Stacked base pairs, bulges and internal loops

Here, we consider the case of a 1-loop, which comprises the case of stacked base pairs, bulges and internal loops. The following cases correspond to each possibility.

**Stacked base pair**

In this case, (*i,j*) stacks on the base pair (*i*+1,*j*−1), and the partition function *Z* ^∗^(*i*+1,*j*−1;*U,V*) has been computed. Let *stack*(*X,Y,U,V*) denote the free energy of base stack $\begin {array}{ll} 5'-\text {\texttt {XU}}-3'\\ 3'-\text {\texttt {YV}}-5'\\ \end {array}$ obtained by table look-up.

17 $$ \begin{aligned} Z^{*}(i,j;X,Y) &= e^{\left(-\frac{e^{I}_{AU}(X,Y)}{RT}\right)} \cdot \sum_{UV \in \mathcal{B}} \exp\left(- \frac{stack(X,Y,U,V)}{RT}\right)\\ &\quad\times Z^{*}\left(i+1,j-1,U,V\right) \end{aligned}  $$

**Bulge loop**

In this case, (*i,j*) closes a bulge in *s*_0_. Since bulge size may exceed the values in table look-up, we define the free energy for a bulge of size *r* by

18 $$\begin{array}{*{20}l} bulge(r) &= \left\{ \begin{array}{ll} bulgeE(r) &\text{if~} r \leq 30\\ bulgeE(30)+1.75 RT \ln\left(\frac{r}{30} \right) &\text{otherwise.}\\ \end{array} \right. \end{array} $$

If (*i,j*) closes a left bulge of size *r* in *s*_0_, then the bulge is closed by base pair (*i*+*r*+1,*j*−1) involving nucleotide pair *U,V*, and

19 $$ \begin{aligned} Z^{*}(i,j;X,Y) &= e^{\left(-\frac{e^{I}_{AU}(X,Y)}{RT}\right)} \cdot \sum_{UV \in \mathcal{B}} \exp\left(-\frac{e_{AU}\left(i,j,X,Y\right)}{RT}\right) \\ &\quad\times\exp\left(- \frac{bulge(r)}{RT}\right) \cdot 4^{r} \cdot Z^{*}\left(i+r+1,j-1,U,V\right) \end{aligned}  $$

while if (*i,j*) closes a right bulge in *s*_0_, then the bulge is closed by base pair (*i*+1,*j*−*r*−1) involving nucleotide pair *U,V*, and

20 $$ \begin{aligned} Z^{*}(i,j;X,Y) &= e^{\left(-\frac{e^{I}_{AU}(X,Y)}{RT}\right)} \cdot \sum_{UV \in \mathcal{B}} \exp\left(-\frac{e_{AU}(i,j,X,Y)}{RT}\right)\\ &\quad\times\exp\left(- \frac{bulge(r)}{RT}\right) \cdot 4^{r} \cdot Z^{*}\left(i+1,j-r-1,U,V\right) \end{aligned}  $$

**Internal loop**

In this case, (*i,j*) closes an internal loop in *s*_0_, whose left [resp. right] portion is of size *r*_1_ [resp. *r*_2_]. Since internal loop size *r*=*r*_1_+*r*_2_ may exceed the values in table look-up, we define the free energy for an internal loop of size *r* by

21 $$\begin{array}{*{20}l}  internal(r) = \!\left\{\! \begin{array}{ll} internalE(r) &\text{if~} r \leq 30\\ internalE(30)+1.75 RT \ln\left(\frac{r}{30} \right) &\text{otherwise.}\\ \end{array} \right. \end{array} $$

The closing base pair (*i*+*r*_1_+1,*j*−*r*_2_−1) of the internal loop of size *r*=*r*_1_+*r*_2_ may involve the nucleotides $UV \in \mathcal {B}$, while the unpaired (mismatch) nucleotides in positions *i*+1,*j*−1,*i*+*r*_1_,*j*−*r*_2_ may involve $A,B,C,D \in \mathcal {N}$. In addition, there is an energy penalty for non symmetric internal loops, *min*(*asym*·|*r*_1_−*r*_2_|,*maxAsym*), where the value of the constants *asym* and *maxAsym* are given in the Turner energy model. Thus

$$\begin{aligned} Z^{*}(i,j;X,Y) &= e^{\left(-\frac{e^{I}_{AU}(X,Y)}{RT}\right)} \cdot \exp\left(-\frac{min\left(asym \cdot |r_{1} - r_{2}|, maxAsym\right)}{RT}\right) \\ &\quad\times \sum_{UV \in \mathcal{B}} \sum_{A,B,C,D \in \mathcal{N}} \exp\left(-\frac{e_{AU}\left(i,j,X,Y\right)}{RT}\right)\\ &\quad\times\exp\left(- \frac{internal\left(r_{1}+r_{2}\right)}{RT}\right) \cdot 4^{r_{1}+r_{2}-4} \\ &\quad\times\exp\left(- \frac{mismatch(X,Y,A,B)+mismatch(V,U,D,C)}{RT}\right) \end{aligned} $$

22 $$ \begin{aligned} \;\;\quad\qquad \times Z^{*}\left(i+r_{1}+1,j-r_{2}-1,U,V\right) \end{aligned}  $$

#### External loop

Despite the fact that, by following the total order on base pairs defined in Eq. (10), the *dual partition function* of multiloops is always computed before the *dual partition function* of the external loop, the computation of the *dual partition function* of multiloops will be easier to understand if the *dual partition function* of the external loop is defined in advance.

In order to improve speed, some implementations of RNA thermodynamics-based algorithms ignore the contribution of dangling positions, which corresponds to Vienna RNA Package -d0 flag. RNAdualPF also includes this option, which dramatically increases the speed of the algorithm. The reason behind this difference of performance is clear from the following definitions.

Suppose that *H*=[(*i*_1_,*j*_1_),…,(*i*_*k*_,*j*_*k*_)] constitutes the list of *k* external base pairs of *s*_0_, where *i*_1_<*j*_1_<*i*_2_<*j*_2_< ⋯ < *i*_*k*_ < *j*_*k*_. For each (*i*_*r*_,*j*_*r*_), with 1≤*r*≤*k*, and for each choice of base pair GC, CG, AU, UA, GU, UG, the value *Z* ^∗^(*i*_*r*_,*j*_*r*_;*X*_*r*_,*Y*_*r*_) has been previously computed and stored by dynamic programming, as well as the sum *Z* ^∗^(*i*_*r*_,*j*_*r*_). When the contribution of dangles is ignored, the *dual partition function* of an external loop with *ℓ* nucleotide positions external to every base pair is defined by

23 $$\begin{array}{*{20}l}  Z^{*}(s_{0}) &= 4^{\ell} \cdot \prod\limits_{r=1}^{k} Z^{*}(i_{r},j_{r}) \end{array} $$

where $\ell = n-\sum \limits _{r = 1,\ldots,k}{(j_{r}-i_{r}+1)}$ and *n* is the length of the target structure *s*_0_.

The default treatment of dangles in RNAdualPF described below corresponds to Vienna RNA Package -d2 flag, where both flanking positions of each external base pair contribute to the free energy. Let *D*=[*a*_1_,*b*_1_,…,*a*_*k*_,*b*_*k*_]⊆[*i*_1_−1,*j*_1_+1,⋯,*i*_*k*_,*j*_*k*_] be a list of those nucleotide positions that are adjacent to the *k* external base pairs (*i*_1_,*j*_1_),…,(*i*_*k*_,*j*_*k*_). The ordered multiset [*a*_1_,*b*_1_,…,*a*_*k*_,*b*_*k*_] can be considered as a collection of constraints, so that (for instance) if *a*_2_=*i*_2_−1, and *a*_2_=*j*_1_+1, then *a*_2_=*b*_1_ and any nucleotide value that is assigned to *b*_1_ must simultaneously be assigned to *a*_2_. Moreover, there can also be an overlap between the list of base paired positions in *H* [*i*_1_,*j*_1_,…,*i*_*k*_,*j*_*k*_] and the multiset *D*=[*a*_1_,*b*_1_,…,*a*_*k*_,*b*_*k*_]. If (for instance) *j*_1_=*i*_2_−1, then *b*_1_=*i*_2_ and *a*_2_=*j*_1_. Therefore, in the computation we have to account for these constraints. Let *m* denote the number of unpaired positions in *D*, without repetitions, and define *A*_*r*_,*B*_*r*_ as the nucleotides instantiated respectively at *a*_*r*_,*b*_*r*_. The energy term for a 5^′^-dangle [resp. 3^′^-dangle] on base pair (*X,Y*) with nucleotides *U,V* is denoted by *E*_*d*5_(*X,Y,x*−1;*U,V,W*) [resp *E*_*d*3_(*X,Y,y*+1;*U,V,W*)] where the dangle position *x*−1 [resp. *y*+1] is assigned nucleotide *W*. With the notation just described, we have

24 $$ \begin{aligned} Z^{*}(s_{0}) &= \sum\limits_{\langle \left(U_{1},V_{1}\right),\ldots,\left(U_{k},V_{k}\right) \rangle \in \mathcal{B}^{k}} \sum\limits_{\left\{A_{1},B_{1},\ldots,A_{k},B_{k} \in \mathcal{N}^{2k}\right\}} 4^{\ell-m} \\ &\quad\times\prod\limits_{r=1}^{k} \left(Z^{*}\left(i_{r},j_{r};U_{r},V_{r}\right)\right.\\ &\quad\times\exp \left(-\frac{E_{d5}\left(i_{r},j_{r},a_{r};U_{r},V_{r},A_{r}\right) +E_{d3}\left(i_{r},j_{r},b_{r};U_{r},V_{r},B_{r}\right)}{RT} \right) \end{aligned}  $$

Depending on the target structure *s*_0_, it can happen that the second sum of Eq. (24) must be restricted to range over strictly less than 4^2*k*^ many RNA sequences. This is explained as follows. If *i*_1_=1 [resp. *j*_*r*_=*n*] then there is no position for a 5^′^ [resp. 3^′^] dangle, and hence the nucleotide sequences considered in the second summation would have length strictly less than 2*k*. Moreover, certain 5^′^ dangled positions could be identical to 3^′^ dangle positions, which arises for instance when *j*_*k*_+2=*i*_*k*+1_; alternatively, certain dangled positions could be identical with base-paired positions, which arises for instance when *j*_*k*_+1=*i*_*k*+1_. In such situations, instantiations of the 3^′^-dangle on (*i*_*k*_,*j*_*k*_) and the 5^′^-dangle on (*i*_*k*+1_,*j*_*k*+1_) are not independent, thus leading to a restriction of the range of the second summation in Eq. (24). A similar restriction is implicitly assumed in the treatment of external loops in this section and of multiloops in the next section.

The algorithm performance can be improved by dividing the external loop into groups of components having interdependently constrained dangling positions, as just explained. Define two base pairs (*X,Y*),(*x* ^′^,*y* ^′^) as adjacent if *x*<*y*<*x* ^′^<*y* ^′^ and *x* ^′^−*y*≤2 – i.e. dangling positions of the base pairs (*X,Y*),(*x* ^′^,*y* ^′^) are constrained. Let *G* denote a *maximal* collection of *adjacent* base pairs belonging to *H*=[(*i*_1_,*j*_1_),…,(*i*_*k*_,*j*_*k*_)], together with their associated dangle positions in *D*=[*i*_1_−1,*j*_1_+1,…,*i*_*k*_−1,*j*_*k*_+1]. It is important to note that *H*∪*D* is thus partitioned into a collection of *g* disjoint groups $\mathcal {G} = [G_{1},\ldots,G_{g}]$. Therefore, we can divide an external loop of *k* helices into a collection groups $\mathcal {G}$ of size *g*≤*k*, and *p* unpaired positions that are external to every base pair of *s*_0_ and not adjacent to any base pair.

For a group *G* with *h* base pairs, let *H*(*G*)=[(*κ*_1_,*λ*_1_),…,(*κ*_*k*_,*λ*_*k*_)] denote the list of base pairs in *G*, and let *D*(*G*)=[*α*_1_,*β*_1_,…,*α*_*h*_,*β*_*h*_]⊆[*κ*_1_−1,*λ*_1_+1,⋯,*κ*_*h*_−1,*λ*_*h*_+1] denote their associated dangle positions. If *U*_*r*_,*V*_*r*_,*A*_*r*_,*B*_*r*_ denote the nucleotides instantiated at the base pair *r*=(*κ*_*r*_,*λ*_*r*_) and its respective dangling positions *α*_*r*_,*β*_*r*_ respectively, then the *dual partition function* of *G* is the following.

25 $$ \begin{aligned} Z^{*}(G) &=\sum\limits_{\langle (U_{1},V_{1}),\ldots,(U_{h},V_{h}) \rangle \in \mathcal{B}^{h}} \sum\limits_{\left\{A_{1},B_{1},\ldots,A_{h},B_{h} \in \mathcal{N}^{2h}\right\}} \\ &\quad\times\prod\limits_{r=1}^{h} \left(Z^{*}\left(\kappa_{r},\lambda_{r};U_{r},V_{r}\right)\right.\\ &\quad\times\exp\left(-\frac{E_{d5}\left(\kappa_{r},\lambda_{r},\alpha_{r};U_{r},V_{r},A_{r}\right) +E_{d3}\left(\kappa_{r},\lambda_{r},\beta_{r};U_{r},V_{r}B_{r}\right)}{RT} \right) \end{aligned}  $$

where the range of the second summation can be constrained by the overlap among positions in *D*(*G*) and between positions in *D*(*G*) and *H*(*G*), as explained for Eq. (24).

Finally, since there are no shared dangling positions between groups, the *dual partition function* of an external loop is defined by

26 $$\begin{array}{*{20}l}  Z^{*}(s_{0}) = 4^{p} \cdot \prod\limits_{r=1}^{g} Z^{*}(G_{r}). \end{array} $$

#### Multiloop

Suppose that (*i,j*) closes a multiloop in *s*_0_, which is a *k*-loop, or (*k*+1)-way junction, for *k*>1, where there are *ℓ* unpaired bases in the multiloop. Suppose that the *k* components of the multiloop are closed by the base pairs (*i*_1_,*j*_1_),…,(*i*_*k*_,*j*_*k*_) with the property that *i*<*i*_1_<*j*_1_<*i*_2_<*j*_2_<⋯<*i*_*k*_<*j*_*k*_<*j*. Assume that for all nucleotide choices in $\mathcal {B}$ for each of the *k* base pairs of the multiloop (*i*_*r*_,*j*_*r*_), for 1≤*r*≤*k*, the value *Z* ^∗^(*i*_*r*_,*j*_*r*_;*X*_*r*_,*Y*_*r*_) has previously been computed and stored by dynamic programming, as well as the sum *Z* ^∗^(*i*_*r*_,*j*_*r*_). The computation of the *dual partition function* is similar to that of the external loop. However, in this case we have to add the contribution of the base pair closing the multiloop (*i,j*), the AU-penalties applied to this base pair, and the energetic penalty of a multiloop *a*+*b*·(*k*+1)+*c*·*ℓ*, where the values of the constants *a*, *b* and *c* are given in the Turner energy model. Then, the *dual partition function* of a multiloop without accounting for dangling positions is

27 $$ \begin{aligned} Z^{*}(i,j;X,Y) &= e^{\left(-\frac{e^{I}_{AU}(X,Y)}{RT}\right)} \cdot \exp\left(-\frac{a+b\cdot(k+1)+c\ell}{RT}\right) \cdot 4^{\ell} \\ &\quad\times\exp\left(-\frac{e_{AU}(i,j,X,Y)}{RT}\right) \cdot \sum\limits_{\langle \left(U_{1},V_{1}\right),\ldots,\left(U_{k},V_{k}\right) \rangle \in \mathcal{B}^{k}}\\ &\quad\times\prod\limits_{r=1}^{k} Z^{*}\left(i_{r},j_{r};U_{r},V_{r}\right) \end{aligned}  $$

The notation we use to define the *dual partition function* of multiloops with dangling positions is similar to that described for external loops. However, some modifications are required in the previously given definitions, since we have to take into account the flanking positions of the base pair (*i,j*) closing the multiloop. Let *H*=[(*i*_1_,*j*_1_),…,(*i*_*k*_,*j*_*k*_),(*i,j*)] be the collection of *k* base pairs closing one of the *k* components of the multiloop, and the base pair (*i,j*) closing the multiloop, and define the multiset *D*=[*a*_1_,*b*_1_,…,*a*_*k*+1_,*b*_*k*+1_]⊆[*i*_1_−1,*j*_1_+1,⋯,*i*_*k*_−1,*j*_*k*_+1,*i*+1,*j*−1] of nucleotide positions adjacent to the base pairs in *H*. Due to the possible overlap with the base pair closing the multiloop and its flanking positions, there are additional constraints in the ordered multiset [*a*_1_,*b*_1_,…,*a*_*k*+1_,*b*_*k*+1_], so that (for instance) if *a*_1_=*i*_1_−1, and *i*_1_=*i*+1, then *a*_1_=*a*_*k*+1_ and any nucleotide value that is assigned to *a*_1_ must simultaneously be assigned to *a*_*k*+1_. Moreover, there can also be an overlap between the list of base paired positions [*i*_1_,*j*_1_,…,*i*_*k*_,*j*_*k*_,*i,j*] and the multiset [*a*_1_,*b*_1_,…,*a*_*k*+1_,*b*_*k*+1_]. If (for instance) *i*=*i*_1_−1, then *a*_*k*+1_=*i*_1_ and *a*_1_=*i*.

Let *m* denote the number of unpaired positions in *D*, without repetitions. Then, the *dual partition function* of a multiloop with dangling positions is defined as follows.

28 $$ \begin{aligned} Z^{*}(i,j;X,Y) &= e^{\left(-\frac{e^{I}_{AU}(X,Y)}{RT}\right)} \cdot \sum\limits_{\langle \left(U_{1},V_{1}\right),\ldots,\left(U_{k},V_{k}\right) \rangle \in \mathcal{B}^{k}} \sum\limits_{\left\{A_{1},B_{1},\ldots,A_{k+1},B_{k+1} \in \mathcal{N}^{2(k+1)}\right\}} \\ &\quad\exp\left(-\frac{a+b\cdot(k+1)+c\ell}{RT}\right) \cdot 4^{\ell-m} \cdot \exp\left(-\frac{e_{AU}\left(i,j,X,Y\right)}{RT}\right) \\ &\quad\times\prod\limits_{r=1}^{k} \left({\vphantom{\left.\quad\times\exp(-\frac{E_{d5}\left(i_{r},j_{r},a_{r};U_{r},V_{r},A_{r}\right) +E_{d3}(i_{r},j_{r},b_{r};U_{r},V_{r},B_{r})}{RT} \right)}}Z^{*}\left(i_{r},j_{r};U_{r},V_{r}\right)\right.\\ &\left.\quad\times\exp(-\frac{E_{d5}\left(i_{r},j_{r},a_{r};U_{r},V_{r},A_{r}\right) +E_{d3}(i_{r},j_{r},b_{r};U_{r},V_{r},B_{r})}{RT} \right) \\ & \quad\times\exp\left(-\frac{E_{d3}\left(j,i,a_{k+1};Y,X,A_{k+1}\right) +E_{d5}\left(j,i,b_{k+1};Y,X,B_{k+1}\right)}{RT}\right) \end{aligned}  $$

As explained for Eq. (24), it can happen that the second summation must be restricted to range over strictly less than 4^2*k*^ many RNA sequences.

A decomposition similar that for external loops can be performed to improve the performance in the computation of the *dual partition function* of a multiloop. In a multiloop, in addition to the adjacency definition given for external loops, we consider the base pair (*i,j*) that closes the multiloop as adjacent to a base pair (*X,Y*) that closes a component of the multiloop, where *i*<*x*<*y*<*j*, if either *x*≤*i*+2 or *y*≥*j*−2. Then, let G denote a *maximal* collection of *adjacent* base pairs belonging to *H*=[(*i*_1_,*j*_1_),…,(*i*_*k*_,*j*_*k*_),(*i,j*)], together with their associated dangle positions in *D*=[*i*_1_−1,*j*_1_+1,…,*i*_*k*_−1,*j*_*k*_+1,*i*+1,*j*−1]. This decomposition produces a collection $\mathcal {G}$ of *g* disjoint groups *G*_1_,…,*G*_*g*_, one of which, designated the *closing group* *G*_*c*_ contains the closing base pair (*i,j*) of the multiloop, and *g*−1 of which, designated as *non-closing groups* *G*_*nc*_, do not contain the base pair (*i,j*).

*Non-closing groups* have the same composition as those defined for external loops – i.e. a collection of *h* base pairs *H*(*G*_*nc*_)=[(*κ*_1_,*λ*_1_),…,(*κ*_*h*_,*λ*_*h*_)] and a set of dangling positions *D*(*G*_*nc*_)=[*α*_1_,*β*_1_,…,*α*_*h*_,*β*_*h*_]⊆[*κ*_1_−1,*λ*_1_+1,⋯,*κ*_*h*_−1,*λ*_*h*_+1]. Therefore, we can compute the *dual partition function* *Z*(*G*_*gc*_) of a *non-closing group* as described in Eq. (25). In addition, the collection of *non-closing groups* of size *g*−1 of a multiloop of *k* components is denoted by $\mathcal {G}_{nc}$, where 0≤(*g*−1)≤*k*.

Therefore, a multiloop of *k* components and *ℓ* unpaired positions can be decomposed into one closing group *G*_*c*_, a collection of non-closing groups $\mathcal {G}_{nc}$, and *p* unpaired positions that are not adjacent to any base pair, with 0≤*p*≤*ℓ*.

**Fig. 1:**
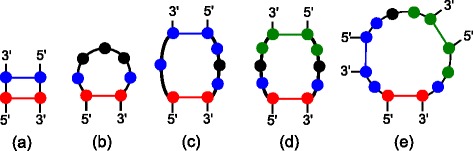
Sampling dependency examples in RNAdualPF for different structural elements: (**a**) stacked base pair, (**b**) hairpin, (**c**) 1×3 internal loop, (**d**) 3×3 internal loop and (**e**) multiloop. Base pair (*i,j*) to be sampled is highlighted in *red*, positions whose energy contribution is dependent on the instantiation of (*i,j*) are highlighted in *blue*, and positions that are mutually dependent, but independent of the instantiation of (*i,j*), are highlighted in *green*. Unpaired positions where the nucleotide choice has no effect in the free energy of the structure are indicated in *black*

In a *non-closing group*, the collection of base pairs of size *h*+1 is denoted by *H*(*G*_*c*_)=[(*κ*_1_,*λ*_1_),…,(*κ*_*h*_,*λ*_*h*_),(*i,j*)], where the base pair (*i,j*) closing the multiloop is at the last position. The ordered multiset of adjacent positions is denoted by *D*(*G*_*c*_)=[*α*_1_,*β*_1_,…,*α*_*h*+1_,*β*_*h*+1_]⊆[*κ*_1_−1,*λ*_1_+1,⋯,*κ*_*h*_−1,*λ*_*h*_+1,*i*+1,*j*−1], where the positions adjacent to *i* and *j* are at the last positions are respectively denoted by *α*_*h*+1_,*β*_*h*+1_. A graphical example of a *closing group* and a *non-closing group* is shown in Fig. 1 e, where the positions of a *non-closing group* with 1 base pair are highlighted in green and the positions of the *closing group* are highlighted in red and blue, and where the base pair (*i,j*) that closes the multiloop is depicted in red.

For a *closing group* *G*_*c*_ with *h*+1 base pairs in *H*(*G*_*c*_)=[(*κ*_1_,*λ*_1_),…,(*κ*_*h*_,*λ*_*h*_),(*i,j*)] and their flanking positions *D*(*G*_*c*_)=[*α*_1_,*β*_1_,…,*α*_*h*+1_,*β*_*h*+1_]⊆[*κ*_1_−1,*λ*_1_+1,⋯,*κ*_*h*_−1,*λ*_*h*_+1,*i*+1,*j*−1], let *X,Y* denote the nucleotides assigned to the closing base pair of the multiloop (*i,j*), and let *U*_*r*_,*V*_*r*_,*A*_*r*_,*B*_*r*_ denote the nucleotides assigned respectively to the base pair *r*=(*κ*_*r*_,*λ*_*r*_,) and its flanking positions *α*_*r*_,*β*_*r*_. Then, the the *dual partition function* *Z* ^∗^(*G*_*c*_;*X,Y*) of the *closing group* is defined by

29 $$ \begin{aligned} e^{\left(-\frac{e^{I}_{AU}(X,Y)}{RT}\right)} &\cdot \sum\limits_{\langle \left(U_{1},V_{1}\right),\ldots,\left(U_{k},V_{h}\right) \rangle \in \mathcal{B}^{h}} \sum\limits_{\left\{A_{1},B_{1},\ldots,A_{h+1},B_{h+1} \in \mathcal{N}^{2(h+1)}\right\}}\\ &\quad\times\exp\left(-\frac{e_{AU}\left(i,j,X,Y\right)}{RT}\right) \cdot\prod\limits_{r=1}^{h} \left(Z^{*}\left(\kappa_{r},\lambda_{r};U_{r},V_{r}\right)\right.\\ &\times\exp\left(-\frac{E_{d5}\left(\kappa_{r},\lambda_{r},\alpha_{r};U_{r},V_{r},A_{r}\right) +E_{d3}\left(\kappa_{r},\lambda_{r},\beta_{r};U_{r},V_{r},B_{r}\right)}{RT} \right) \\ & \times\exp\left(-\frac{E_{d3}\left(j,i,\alpha_{h+1};Y,X,A_{h+1}\right) +E_{d5}\left(j,i,\beta_{h+1};Y,X,B_{h+1}\right)}{RT}\right) \end{aligned}  $$

In the same way as in Eq. (24), the values of the second summation are constrained to the possible choices among overlapping positions.

Then, the *dual partition function* *Z* ^∗^(*i,j*;*X,Y*) of the multiloop with *k* components and *ℓ* unpaired positions, where *p* of which are not adjacent to any base pair, is defined by

30 $$\begin{array}{*{20}l}  Z^{*}(i,j;X,Y) &= \exp\left(-\frac{a+b\cdot(k+1)+c\ell}{RT}\right) \cdot 4^{p}\\ & \quad\times Z^{*}\left(G_{c};X,Y\right) \cdot \prod\limits_{G_{nc} \in \mathcal{G}_{nc}} Z^{*}\left(G_{nc}\right)  \end{array} $$

### Sampling

**Fig. 2:**
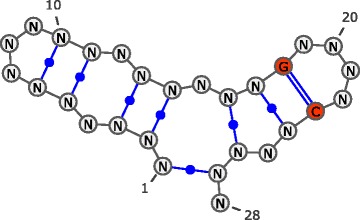
Target structure with sequence constraints used as input of RNAdualPF to compute the *dual partition function* values shown in Table 1. Sequence constraints are highlighted in *red*

Once the *dual partition function* *Z* ^∗^(*i,j*) and its subcases *Z* ^∗^(*i,j*;*X,Y*) for each base pair (*i,j*) have been computed, it is possible to perform a Boltzmann weighted sampling of positions *i* and *j*. For example, given the target structure with sequence constraints depicted in Fig. 2, RNAdualPF computes the *dual partition function* table shown in Table 1. The *dual partition function* of the substructure enclosed by the base pair (*i,j*) is *Z* ^∗^(*i,j*), and the *dual partition function* of the substructure enclosed by the base pair (*i,j*) where *i,j* are currently instantiated by the nucleotides *X,Y* is denoted by is *Z* ^∗^(*i,j*;*X,Y*). Therefore, the Boltzmann probability of *X,Y* at positions *i,j* in the substructure enclosed by the base pair (*i,j*) is *Z* ^∗^(*i,j*;*X,Y*)/*Z* ^∗^(*i,j*) and can be sampled using the roulette wheel method.

Due to the Turner energy model, it is necessary to determine nucleotide positions whose instantiation influences the energy (hence Boltzmann probability) of other positions, and subsequently all mutually dependent positions must be instantiated simultaneously. Figure 1 illustrates the mutual dependencies that must be considered when sampling different types of elements, where the base pair (*i,j*) to be sampled is highlighted in red, positions whose sampling probability is dependent on the instantiation of (*i,j*) are highlighted in blue, and positions that are mutually dependent, but independent of the instantiation of (*i,j*), are highlighted in green.

Since the dynamic programming algorithm for the *dual partition function* proceeds from inner to outer base pairs, using the total ordering ≺ in Eq. (10), the sampling order of base pairs proceeds from outer to inner positions, i.e. from largest *base pair index* to smallest. In order to account for mutual dependencies in the sampling step, we define the function *sample*(*k,T,i,j,X,Y*) for each base pair (*i,j*) in *S*_0_, where *k* indicates the *base pair index* defined from Eq. (10), *T* indicates the type of structural element closed by base pair (*i,j*) in the target RNA secondary structure, as shown in Table 1, and *X,Y* are the instantiated nucleotides at positions (*i,j*). Due to the mutual dependencies, sampling a base pair with *base pair indexk* closing an *m*-loop, for *m*>0, forces the instantiation of all inner closing base pairs of the *m*-loop, and the *base pair index* of each such inner base pair is strictly less than *k*. For this reason, except in the case of external loops, the outermost base pair (*i,j*) has been always instantiated before *sample*(*k,T,i,j,X,Y*) is called, and therefore the instantiation *X,Y* is given as a parameter of the sampling function.

The Boltzmann probability of each possible instantiation of mutually dependent positions can be computed on the fly in the backward step. However, in order to improve the speed of the algorithm, in the forward step RNAdualPF stores (for each base pair) the conditional *dual partition function* values of instantiations of interdependent positions. These tables are used by the sampling function, since each value corresponds to the *dual partition function* conditional on a specific instantiation of the positions to be sampled by *sample*(*k,T,i,j,X,Y*). Since the sampling procedure depends on the type *T* of element, we describe the function *sample*(*k,T,i,j,X,Y*) for each type of element – hairpin, stacked base pair, internal loop (which also comprises left and right bulge), multiloop and external loop as depicted in Fig. 1. For each of these cases, the values are stored in the conditional *dual partition function* table associated with the closing base pair (*i,j*).

#### Hairpins

When hairpin size exceeds three (Fig. 1 a), since the base pair (*i,j*) has been previously instantiated, flanking positions *i*+1,*j*−1 are sampled first. Given the current assignment *X,Y*, the Boltzmann probability of sampling respectively the nucleotides *U,V* at the flanking positions *i*+1,*j*−1 is

31 $$\begin{array}{*{20}l}  P&\left(i+1=U,j-1=V|i=X,j=Y\right)\\ &=\frac{Z^{*}\left(i,j,i+1,j-1;X,Y,U,V\right)}{Z^{*}\left(i,j;X,Y\right)} \end{array} $$

Therefore, in the forward step RNAdualPF stores in a table the conditional *dual partition function* of each possible instantiation {*X,Y,U,V*} of the base pair (*i,j*) and its flanking positions *i*+1,*j*−1 respectively, defined by

32 $$\begin{array}{*{20}l} &Z^{*}\left(i,j,i+1,j-1;X,Y,U,V\right)\\ &\quad= e^{\left(-\frac{e^{I}_{AU}(X,Y)}{RT}\right)} \cdot \exp\left(- \frac{H(j-i-1)}{RT} \right) \cdot \\ &\qquad\exp\left(- \frac{mismatch(X,Y,U,V)}{RT} \right) \cdot 4^{j-i-3} \end{array} $$

Then, remaining unpaired positions are uniformly sampled, since the nucleotide choice does not change the final free energy. Triloops, tetraloops and hexaloops are exceptions to this rule, since there are special loops that contribute to or penalize the free energy. In those cases, we have to account for the special loops, as defined in “Hairpins” section.

Although it could seem to be a waste of space to store a different conditional *dual partition function* table for each base pair (*i,j*), even for two different hairpins of the same size in the target structure, one should note that RNAdualPF allows sequence constraints, and thus *Z* ^∗^(*i,j*) could possibly differ from *Z* ^∗^(*i* ^′^,*j* ^′^) when (*i,j*) and (*i* ^′^,*j* ^′^) close hairpins of the same size.

#### Stacking base pairs

As depicted in Fig. 1 b, sampling probability of a base pair with *base pair index* *k*−1 is dependent on the value sampled at the adjacent stacking base pair with *base pair indexk*. Therefore, *sample*(*k,Stack,i,j,X,Y*) samples the base pair (*i*+1,*j*−1) using the conditional probability given the instantiation of base pair (*i,j*) by *X,Y*, defined as follows:

33 $$\begin{array}{*{20}l} P&\left(i+1=U,j-1=V|i=X,j=Y\right) \\ &= \frac{Z^{*}\left(i,j,i+1,j-1;X,Y,U,V\right)}{Z^{*}\left(i,j;X,Y\right)} \end{array} $$

The conditional *dual partition function* values stored in the forward step correspond to each instantiation {*X,Y,U,V*} of the base pairs (*i,j*),(*i*+1,*j*−1), denoted by

34 $$\begin{array}{*{20}l} &Z^{*}\left(i,j,i+1,j-1;X,Y,U,V\right)\\ &\quad= e^{\left(-\frac{e^{I}_{AU}(X,Y)}{RT}\right)} \cdot \exp\left(- \frac{stack(X,Y,U,V)}{RT}\right)  \\ &\qquad\times Z^{*}\left(i+1,j-1,U,V\right) \end{array} $$

#### Internal loops

The energy contribution of internal loops in the Turner energy model depends on the flanking unpaired positions of both the inner and outer closing base pairs, hence the sampling probability of the inner base pair cannot be separated from the adjacent unpaired positions. Moreover, for specific sizes of internal loop (1×1, 1×2, 2×1, 1×*N* and *N*×1), the inner and outer closing base pairs share flanking positions. In these cases, all the unpaired positions and the outer base pair must be sampled at the same time, since the energy contribution of each combination of base pairs and flanking positions is different. In the 1×3 internal loop depicted in Fig. 1 c, if the outer base pair (*i,j*) is instantiated by *X,Y*, then let *P*(*k*=*U,l*=*V,n*_1_=*A,n*_2_=*B,n*_3_=*C*|*i*=*X,j*=*Y*) denote the probability of sampling the nucleotides *U,V,A,B,C* respectively at positions *k,l,n*_1_,*n*_2_,*n*_3_, where (*k,l*) is the inner closing base pair, *n*_1_ is the flanking position at *i*+1 shared by the base paired positions *i* and *k*, and *n*_2_ and *n*_3_. In the following equation, let *P*(*U,V,A,B,C*|*X,Y*) abbreviate the conditional probability just defined. Then

35 $$\begin{array}{*{20}l} &P\left(U,V,A,B,C|X,Y\right)\\ &\quad=\frac{Z^{*}\left(i,j,k,l,n_{1},n_{2},n_{3};X,Y,U,V,A,B,C\right)}{Z^{*}\left(i,j;X,Y\right)} \end{array} $$

RNAdualPF computes and stores the conditional *dual partition function* of each possible instantiation {*X,Y,U,V,A,B,C*} respectively at positions *i,j,k,l,n*_1_,*n*_2_,*n*_3_, where the value *Z* ^∗^(*i,j,k,l,n*_1_,*n*_2_,*n*_3_;*X,Y,U,V,A,B,C*) is defined by

36 $$ \begin{aligned} e^{\left(-\frac{e^{I}_{AU}(X,Y)}{RT}\right)}& \cdot \exp\left(-\frac{min(asym \cdot |(k - i) - (j - l)|, maxAsym)}{RT}\right)\\ &\times 4^{j-l-3} \cdot \exp\left(-\frac{e_{AU}(i,j,X,Y)}{RT}\right)\\ &\times\exp\left(- \frac{internal\left(k-i+j-l-2\right)}{RT}\right) \\ & \times \exp\left(- \frac{mismatch(X,Y,A,B)+mismatch(V,U,C,A)}{RT}\right)\\ &\times Z^{*}(k,l,U,V) \end{aligned}  $$

For internal loops of sizes (1×1, 1×2, 2×1, 1×*N* and *N*×1) similar conditional *dual partition function* tables are computed following the definitions in “Stacked base pairs, bulges and internal loops” section.

**Other internal loops:** When there are no shared flanking positions between the two base pairs that close an internal loop, as depicted in Fig. 1 d, the energy contribution of innermost base pair and its respective flanking positions is independent of those of the outermost base pair.

In this case, RNAdualPF samples first the flanking positions *i*+1,*j*−1 of the outermost base pair (*i,j*), whose sampling probability is solely dependent on the instantiated nucleotides *X,Y* at positions *i,j*. Is not necessary to store any conditional *dual partition function* for sampling these positions, since the probability of sampling the values *A,B* at the flanking positions *i*+1,*j*−1, given the assignment *X,Y* is defined by

37 $$\begin{array}{*{20}l} &P\left(i+1=A,j-1=B|i=X,j=Y\right)\\ &\quad= \frac{\exp\left(- \frac{mismatch(X,Y,A,B)}{RT}\right)}{\sum_{C,D \in \mathcal{N}}\exp\left(- \frac{mismatch(X,Y,C,D)}{RT}\right)} \end{array} $$

where mismatch penalties are obtained from table look-up. Finally, the innermost base pair (*k,l*) and its flanking positions *k*−1,*l*+1 are sampled together. In this case, we need to store an additional value *Z* ^∗^(*k*−1,*l*+1), which is given by

38 $$\begin{array}{*{20}l} &Z^{*}\left(k-1,l+1\right)= \sum_{UV \in \mathcal{B}} \sum_{C,D \in \mathcal{N}}\\ &\quad\exp\left(- \frac{mismatch(V,U,D,C)}{RT}\right) \cdot Z^{*}(k,l,U,V) \end{array} $$

Then, following the same notation, the probability of sampling the nucleotides *V,U,D,C* respectively at positions *k,l,k*−1,*l*+1 is

39 $$\begin{array}{*{20}l} P&\left(k=V,l=U,k-1=D,l+1=C\right)\\ &=\frac{Z^{*}\left(k,l,k-1,l+1;V,U,D,C\right)}{Z^{*}\left(k-1,l+1\right)} \end{array} $$

Therefore, the conditional *dual partition function* of each possible instantiation {*V,U,D,C*} stored in the corresponding table is defined as

40 $$\begin{array}{*{20}l} &Z^{*}\left(k,l,k-1,l+1;V,U,D,C\right) \\&\quad= \exp\left(- \frac{mismatch(V,U,D,C)}{RT}\right)\cdot Z^{*}(k,l,U,V)  \end{array} $$

Finally, since the remaining unpaired position does not contribute to the free energy, it is uniformly sampled.

#### Multiloops and external loops

As explained in “External loop” section, if dangling positions are not included in the computation, sampling an external base pair or the closing base pair (*i,j*) of a multiloop from *Z* ^∗^(*i,j*) is trivial. On the other hand, by including dangling positions in the sampling, there is a dramatic increase in the space complexity of RNAdualPF, albeit the space used is only a constant factor larger. However, the decompositions into groups described in “External loop” and “External loop” sections allow to sample the positions of each group independently.

The example shown in Fig. 1 e depicts a multiloop with two groups: a *non-closing group* *G*_*nc*_ highlighted in green, and a *closing group* *G*_*c*_ highlighted in red and blue, where the closing base pair of the multiloop (*i,j*) is marked in red.

In a *non-closing group* *G*_*nc*_ all base pairs in *H*(*G*_*nc*_) and dangling positions in *D*(*G*_*nc*_) must be sampled together. Therefore, the conditional *dual partition function* of each possible instantiation of nucleotides at the *h* closing pairs in *H*(*G*_*nc*_) and their adjacent positions in *D*(*G*_*nc*_) is stored. Let $\mathcal {U}=\{U_{1},V_{1},\ldots,U_{h},V_{h}\}$ denote an instantiation of the *h* base pairs in *H*(*G*_*nc*_)=[*κ*_1_,*λ*_1_,…,*κ*_*h*_,*λ*_*h*_], and let $\mathcal {W}=\{A_{1},B_{2},\ldots,A_{h},B_{h}\}$ denote an instantiation of the *h* flanking positions in *D*(*G*_*nc*_)=[*α*_1_,*β*_1_,…,*α*_*h*_,*β*_*h*_] in the *non-closing group* *G*_*nc*_. Then, the probability of sampling $\mathcal {U},\mathcal {W}$ is

41 $$\begin{array}{*{20}l} &P(H(G_{nc})=\mathcal{U},D(G_{nc})=\mathcal{W})\\ &\quad= \frac{Z^{*}\left(G,H\left(G_{nc}\right),D\left(G_{nc}\right);\mathcal{U},\mathcal{W}\right)}{Z^{*}(G)} \end{array} $$

Therefore, the conditional *dual partition function* of each instantiation $\mathcal {U},\mathcal {W}$ at *H*(*G*_*nc*_),*D*(*G*_*nc*_), stored in the table of the group, is defined by

42 $$ \begin{aligned} &Z^{*}\left(G,H\left(G_{nc}\right),D\left(G_{nc}\right);\mathcal{U},\mathcal{W}\right)\\ &\quad=\prod\limits_{r=1}^{h} \left({\vphantom{\left.\qquad\times\exp\left(-\frac{E_{d5}\left(\kappa_{r},\lambda_{r},\alpha_{r};U_{r},V_{r},A_{r}\right)+E_{d3} \left(\kappa_{r},\lambda_{r},\beta_{r};U_{r},V_{r},B_{r}\right)}{RT} \right)\right)}}Z^{*}\left(\kappa_{r},\lambda_{r};U_{r},V_{r}\right)\right.\\ &\left.\qquad\times\exp\left(-\frac{E_{d5}\left(\kappa_{r},\lambda_{r},\alpha_{r};U_{r},V_{r},A_{r}\right)+E_{d3} \left(\kappa_{r},\lambda_{r},\beta_{r};U_{r},V_{r},B_{r}\right)}{RT} \right)\right) \end{aligned}  $$

Recall that the base pairs in *H*(*G*_*nc*_) are adjacent. Therefore, due the constraints given by the overlapping positions within *D*(*G*_*nc*_), and between *D*(*G*_*nc*_) and *H*(*G*_*nc*_), explained in “External loop” section, the number of possible instantiations $\mathcal {U},\mathcal {W}$ of *H*(*G*_*nc*_),*D*(*G*_*nc*_) is ≤(6^*h*^·4^*h*+1^).

In a similar way, sampling from the *closing group* *G*_*c*_ closed by the base pair (*i,j*), with *h*+1 base pairs in *H*(*G*_*c*_) and their corresponding flanking positions in *D*(*G*_*c*_) requires us to store the conditional *dual partition function* of each instantiation of nucleotides $\{X,Y,\mathcal {U},\mathcal {W}\}$ respectively at *i,j,H*(*G*_*c*_),*D*(*G*_*c*_), where $\mathcal {U}=\{U_{1},V_{1},\ldots,U_{h},V_{h}\}$ denotes an instantiation of the *h* first base pairs [(*κ*_1_,*λ*_1_),…,(*κ*_*h*_,*λ*_*h*_)] in *H*(*G*_*c*_), $\mathcal {W}=\{A_{1},B_{2},\ldots,A_{h+1},B_{h+1}\}$ denotes an instantiation of the 2·(*h*+1) flanking positions in *D*(*G*_*c*_)=[*α*_1_,*β*_1_,…,*α*_*h*+1_,*β*_*h*+1_], and *X,Y* denotes an instantiation of (*i,j*). The probability of the instantiation $\mathcal {U},\mathcal {W}$, given the nucleotides *X,Y* is

43 $$\begin{array}{*{20}l} &P\left(H(G_{c})=\mathcal{U},D(G_{c})=\mathcal{W} | i=X,j=Y\right)\\ &\quad=\frac{Z^{*}\left(G_{c},i,j,H(G_{c}),D(G_{c});X,Y,\mathcal{U},\mathcal{W}\right)}{Z^{*}\left(G_{c};X,Y\right)} \end{array} $$

Then, the values stored in the table of the closing group correspond to the conditional *dual partition function* of each instantiation $\left \{X,Y,\mathcal {U},\mathcal {W}\right \}$ are given by $Z^{*}(G_{c},i,j,H(G_{c}),D(G_{c});X,Y,\mathcal {U},\mathcal {W})$, which is defined by the following expression:

44 $$ \begin{aligned} &e^{\left(-\frac{e^{I}_{AU}(X,Y)}{RT}\right)} \cdot \exp\left(-\frac{e_{AU}(i,j,X,Y)}{RT}\right) \cdot \prod\limits_{r=1}^{h} \left(\left(Z^{*}\left(\kappa_{r},\lambda_{r};U_{r},V_{r}\right) \right.\right.\\ &\left. \cdot\exp\left(-\frac{E_{d5}\left(\kappa_{r},\lambda_{r},\alpha_{r};U_{r},V_{r},A_{r}\right) +E_{d3}\left(\kappa_{r},\lambda_{r},\beta_{r};U_{r},V_{r},B_{r}\right)}{RT} \right)\right)\\ & \cdot \exp\left(-\frac{E_{d3}\left(j,i,\alpha_{h+1};Y,X,A_{h+1}\right) +E_{d5}\left(j,i,\beta_{h+1};Y,X,B_{h+1}\right)}{RT}\right) \end{aligned}  $$

As a final remark, we would like to recall that all the conditional *dual partition function* values are computed and stored in the forward step at the same time as the *dual partition function*. Therefore, despite the consequent increase of space complexity in the algorithm, the computation of the values required for correct sampling does not involve a greater time complexity.

### Scaling

The sequence partition function *Z* ^∗^(*s*_0_) grows much faster than the usual structure partition function *Z*(**a**), and so *scaling* must be used in the implementation. Let *C*>2 be a user-defined constant. By a slight modification of the previous recursions, we actually compute $Z^{\dag }(i,j;X,Y) = \frac {Z^{*}(i,j;X,Y)}{C^{j-i+1}}$, and hence $Z^{\dag }(s_{0}) = \frac {Z^{*}(s_{0})}{C^{n}}$, where *n* is the length of *s*_0_. For instance, the analogue of Eq. (16) is

45 $$ \begin{aligned} Z^{\dag} &= \frac{Z^{*}\left(i,j;x,y\right)}{C^{j-i+1}} \\ &= e^{\left(-\frac{e^{I}_{AU}(x,y)}{RT}\right)} \cdot \frac{\exp\left(- \frac{H(j-i-1)}{RT} \right)}{C^{j-i+1}} \\ &\quad\times \left(\sum_{n_{1},n_{2} \in \mathcal{N}} \exp\left(- \frac{mismatch(x,y,n_{1},n_{2})}{RT}\right) \cdot 4^{j-i-3} \right) \end{aligned}  $$

and the analogue of Eq. (17) is

46 $$ { \begin{aligned} Z^{\dag} &=\frac{Z^{*}(i,j;X,Y)}{C^{j-i+1}}\\ &= e^{\left(-\frac{e^{I}_{AU}(X,Y)}{RT}\right)} \cdot \frac{1}{2} \cdot \sum_{UV \in \mathcal{B}} \exp\left(- \frac{stack(X,Y,U,V)}{RT}\right)\\ &\quad\times Z^{\dag}\left(i+1,j-1,U,V\right) \end{aligned}}  $$

This modification does not affect properties of sequences sampled from the low energy ensemble, since the same scaling factor appears in both the numerator and denominator of all conditional probabilities. For instance, the analogue of Eq. (31) is

47 $$\begin{array}{*{20}l} &P\left(i+1=U,j-1=V|i=X,j=Y\right)\\ &= \frac{Z^{*}\left(i,j,i+1,j-1;X,Y,U,V\right)}{Z^{*}\left(i,j;X,Y\right)} \\ &= \frac{Z^{\dag}\left(i,j,i+1,j-1;X,Y,U,V\right)}{Z^{\dag}(i,j;X,Y)}  \end{array} $$

### Controlling GC-content

The GC-content of an RNA sequence **a**=*s*_1_,…,*s*_*n*_ is the number of nucleotides that are either G or C. Instead of computing *Z* ^∗^(*i,j*;*X,Y*) and *Z* ^∗^(*s*_0_), we can compute *Z* ^∗^(*i,j*;*X,Y*;*α*) and *Z* ^∗^(*s*_0_,*α*), defined to be the corresponding partition *dual partition functions*, restricted to sequences having GC-content of *α*. Note well that GC-content *α* includes the closing nucleotides *X* and *Y* respectively located at positions *i* and *j*; i.e.

48 $$\begin{array}{*{20}l} Z^{*}\left(i,j;X,Y;\alpha\right) &= \sum_{\substack{a_{i},\ldots,a_{j}, GC(a_{i},\ldots,a_{j})=\alpha\\ a_{i}=X, a_{j}=Y, a_{i+1},\ldots,a_{j-1} \in \mathcal{N}}}\\ &\exp\left(-E\left(a_{i},\ldots,a_{j};s_{0}[i,j]\right)/RT\right) \end{array} $$

where *s*_0_[*i,j*] denotes the restriction of target structure *s*_0_ to the interval [*i,j*]. We describe two particular subcases, to provide the idea of how modifications need to be undertaken.

#### Triloop

Note that the number of RNA *sequences* of length *m* having GC-content of *α* is $\binom {m}{\alpha } \cdot 2^{\alpha } \cdot 2^{m-\alpha } = \binom {m}{\alpha } \cdot 2^{m} \leq 4^{m}$, since *α* selected positions must be either G or C, yielding the term 2^*α*^, while the remaining *m*−*α* positions must be either A or U, yielding the term 2^*m*−*α*^. Assume that *γ*(*XY*)=|{*X,Y*}∩{*G,C*}|=*β*. Then

49 $$ \begin{aligned} Z^{*}\left(i,j;X,Y;\alpha\right) &= e^{\left(-\frac{e^{I}_{AU}(X,Y)}{RT}\right)} \cdot \exp\left(- \frac{H(j-i-1) + e_{AU}(xy)}{RT} \right) \\ &\quad\times \left({\vphantom{\sum_{\substack{abc \in TriLoop_{x,y}\\ \gamma(abc)=\alpha-\beta}}}} \binom{j-i-1}{\left(\alpha-\beta\right)} \cdot 2^{j-i-1} - |TriLoop_{x,y}|\right.\\ &\left.\qquad\;+ \sum_{\substack{abc \in TriLoop_{x,y}\\ \gamma(abc)=\alpha-\beta}} \exp\left(- \frac{triloopE(xabcy)}{RT}\right) \right) \end{aligned}  $$

#### Multiloop and external loop

Assume that (*i,j*) closes a multiloop, which is a (*k*+1)-way junction with *ℓ* unpaired nucleotides. Assume that the ordered multiset of potential dangle positions is *D*=[*a*_1_,*b*_1_,…,*a*_*k*+1_,*b*_*k*+1_], where *a*_*r*_=*i*_*r*_−1 and *b*_*r*_=*j*_*r*_+1 for *r*=1,…,*k*, and *a*_*k*+1_=*i* and *b*_*k*+1_=*j*, and assume that there are *m* unpaired positions that are not adjacent to a base pair in the multiloop. If **r** denotes an RNA sequence of arbitrary length, then let the function *γ*(**r**) denote the GC-count in **r**. Given an assignment of nucleotide base pairs *U*_1_ *V*_1_,…,*U*_*k*_ *V*_*k*_ to (*i*_1_,*j*_1_),…,(*i*_*k*_,*j*_*k*_), where *U*_*r*_ *V*_*r*_∈{*GC,CG,AU,UA,GU,UG*}, and given an assignment *A*_1_,*B*_1_,…,*A*_*k*_,*B*_*k*_ of dangle nucleotides, where $A_{r},B_{r} \in \mathcal {N}$, for *r*=1,…,*k*, we let

50 $$\begin{array}{*{20}l} \gamma(\mathbf{AB}) &= \gamma\left(A_{1},\ldots,A_{k},B_{1},\ldots,B_{k}\right). \end{array} $$

Then the *dual partition function* of a multiloop with a GC-content of *α* is defined by setting *Z* ^∗^(*i,j*;*X,Y*;*α*) equal to the following:

51 $$ \begin{aligned} &e^{\left(-\frac{e^{I}_{AU}(X,Y)}{RT}\right)} \cdot \sum\limits_{\alpha_{1} + \cdots + \alpha_{k} \leq \alpha} \sum\limits_{\{U_{r},V_{r} \in \mathcal{B}: r=1,\ldots,k\}} \sum\limits_{\{A_{1},B_{1},\ldots,A_{k},B_{k} \in \mathcal{N}^{2k}\}} \\ &\quad\exp\left(-\frac{a+b\cdot(k+1)+c\ell}{RT}\right) \cdot \binom{(\ell-m)}{\left(\alpha- \sum_{r=1}^{k} \alpha_{r} - \gamma(\mathbf{AB})\right)} \cdot 2^{\ell}\\ &\quad\times\exp\left(-\frac{e_{AU}(i,j,X,Y)}{RT}\right) \cdot \prod\limits_{r=1}^{k} \left(Z^{*}\left(i_{r},j_{r};U_{r},V_{r};\alpha_{r}\right){\vphantom{\frac{0}{0}}}\right.\\ &\quad\times\left.\exp\left(-\frac{E_{d5}\left(i_{r},j_{r},a_{r};U_{r},V_{r},A_{r}\right) +E_{d3}\left(i_{r},j_{r},b_{r};U_{r},V_{r},B_{r}\right)}{RT} \right.\right) \\ &\quad\times\exp\left(-\frac{E_{d3}\left(j,i,a_{k+1};Y,X,A_{k+1}\right) +E_{d5}\left(j,i,b_{k+1};Y,X,B_{k+1}\right)}{RT}\right. \end{aligned}  $$

Since the modification required in the remaining cases follows similar reasoning as in the treatment of the hairpin and external loop just described, the details for these remaining cases are not given.

An additional challenge of computing the *dual partition function* with GC-content control is the combinatorial problem of efficiently counting the number *N* of instantiations of the external loop, consisting of all positions external to every base pair, with GC-content *k*, where the user can stipulate that certain positions are constrained to contain nucleotides consistent with IUPAC codes. To this end, we implemented the combinatorial algorithm defined in Supplementary Information.

#### Sampling with GC-content

The implementation of sampling with GC-content is performed in a similar manner as described in “Sampling” section, with some notable differences.

First, the sampling function is redefined by *sample*(*k,T,i,j,X,Y,α*), where *k* indicates the *base pair index* in the ordering defined by Eq. (10) for the base pair (*i,j*) that is already instantiated by nucleotide pair *XY*, and *T* designates the type of structural element closed by base pair (*i,j*) in the target RNA secondary structure, as shown in Table 1. The function *sample*(*k,T,i,j,X,Y,α*) instantiates all positions of the loop having outer closing base pair (*i,j*), including its inner closing base pair(s) and which returns the GC-content of the sampled loop. Moreover, the GC-content of the subsequence **a**[*i*+1,*j*−1]=(*a*_*i*+1_,…,*a*_*j*−1_) will be *α* once the entire sequence *a*_1_,…,*a*_*n*_ is sampled.

Second, RNAdualPF stores a conditional *dual partition function* table for each base pair (*i,j*) and GC-content 0 to j-i-1. The function *sample*(*k,T,i,j,X,Y,α*) samples from the conditional *dual partition function* of those sequences which have exactly *α* Gs and Cs strictly between the positions *i* and *j*, thus guaranteeing a GC-content of *α* for the subsequence **a**[*i*+1,*j*−1] once the entire sequence *a*_1_,…,*a*_*n*_ is sampled. Note that *sample*(*k,T,i,j,X,Y,α*) samples only the loop closed by the already instantiated outer base pair (*i,j*), and that *α* is the GC-content of the entire subsequence **a**[*i*+1,*j*+1]=*a*_*i*+1_,…,*a*_*j*−1_ once the algorithm terminates. Only in the case that base pair (*i,j*) closes a hairpin loop will it generally happen that the GC-content of the loop closed by (*i,j*) is equal to *α*.

Let *α* be the user-designated GC-content of sequences **a**=*a*_1_,…,*a*_*n*_ to be sampled from a target secondary structure having *ℓ* base pairs. The following pseudocode describes how to sample sequences **a**=*a*_1_,…,*a*_*n*_, whose GC-content exactly equals *α*. Here, an external loop with *m* components means that there are *m* exterior base pairs (*i*_1_,*j*_1_),…,(*i*_*m*_,*j*_*m*_) such that all positions exterior to these base pairs are unpaired; i.e. each position $r \in \{ 1,\ldots,n\} - \cup _{c=1}^{m} \{ i_{c},\ldots,j_{c} \}$ is unpaired in the target structure.



To clarify how the GC-content is sampled in a statistically rigorous manner, suppose that the user has specified the GC-content to be *α*, and that *L* is the external loop of the target structure *s*_0_ having *m* components, where the *c*th component has external closing base pair (*i*_*c*_,*j*_*c*_). In computing the dual partition function, for all possible choices of non-negative integers *α*_1_,…,*α*_*m*_,*β* that sum to *α* and all 6^*m*^ possible assignments of Watson-Crick or wobble nucleotide pairs *X*_1_,*Y*_1_,…,*X*_*m*_,*Y*_*m*_ to the base pairs (*i*_1_,*j*_1_),…,(*i*_*m*_,*j*_*m*_), the software RNAdualPF has computed the sum of $\sum _{c=1}^{m} Z^{*}_{c}(i_{c},j_{c};X_{c},Y_{c};\alpha _{c})$ plus the Boltzmann factor of the external loop with GC-content *β*. Since the dual partition function *Z* ^∗^(*s*_0_;*α*) is the sum, taken over all values of *α*_1_,…,*α*_*m*_,*β* and all Watson-Crick and wobble pair assignments to the external base pairs, RNAdualPF can then use the roulette wheel method to sample values *α*_1_,…,*α*_*m*_,*β* and *X*_1_,*Y*_1_,…,*X*_*m*_,*Y*_*m*_ in a statistically rigorous manner. Multiloops, and other structural elements, which contain unpaired regions whose sequence does not contribute to the free energy of the structure, are handled in a analogous manner.

## Results

### Robustness and plasticity of *C. elegans* miRNAs and *E. coli* sncRNAs

In [1] Borenstein and Ruppin used version 1.4 of the Vienna RNA Package [7] to generate 1000 RNA sequences per wild type precursor microRNA (pre-miRNA) extracted from the database Rfam 1.0 [19], with the property that each of the 1000 control sequences folded into the wild type pre-miRNA structure – i.e. the minimum free energy (MFE) structure of each of the 1000 control sequences was identical to the MFE structure of the wild type pre-miRNA. Based on these computational experiments, Borenstein and Ruppin asserted that the “structure of miRNA precursor stem–loops exhibits a significantly high level of mutational robustness in comparison with random RNA sequences with similar stem–loop structures”. Noting that the Vienna RNA Package inverse folding program RNAinverse does not control for GC-content or other sequence compositional bias, the authors performed a second computational experiment, in which control sequences not only folded into the target wild type structure, but also had similar dinucleotide composition to that of wild type pre-miRNA (Jensen-Shannon divergence less than 0.01). Since the filtering step required enormous run time and computational resources, the authors restricted their attention to a small set of 211 microRNAs, and generated only 100 control sequences per microRNA – note here that RNAinverse cannot control for GC-content. Borenstein and Ruppin concluded that robustness of precursor microRNAs was not the byproduct of a base composition bias or of thermodynamic stability.

Subsequently Rodrigo et al. [3] undertook a similar analysis for bacterial small noncoding RNAs (sncRNA), also using the program RNAinverse, albeit using somewhat different definitions – see precise definitions in “Formal definitions of robustness” section. The main finding of [3] was that bacterial sncRNAs are *not significantly robust* when compared with 1000 sequences having the same structure, as computed by RNAinverse; however, the authors found that bacterial sncRNAs tend to be significantly *plastic*, in the sense that the ensemble of low energy structures are structurally diverse. Unlike the case of precursor microRNAs [1], Rodrigo et al. did not control for sequence compositional bias.

Using RNAdualPF, we performed similar computational experiments on 250 precursor microRNAs of *C. elegans* from miRBase 20 [11] and for the bacterial small noncoding RNAs of [3]. Below, we discuss each case separately.

For each *C. elegans* pre-miRNA, we used RNAdualPF to sample 2000 sequences with no control over GC-content and 2000 sequences whose GC-content was identical with that of the wild type pre-miRNA. Moreover, each control sequence *approximately* folded into the MFE wild type pre-miRNA structure as computed by Vienna RNA Package 2.1.9 [2]. Table 2 shows that length-normalized base pair distance between the MFE structure of the control sequence and that of the pre-miRNA is on average 0.09±0.04 for default use of RNAdualPF with control over GC-content, and 0.06±0.03 when GC-content of each control sequence is identical to that of the corresponding wild type pre-miRNA. Additional measures in Table 2 show that sequences sampled from RNAdualPF (1) are only modestly more stable thermodynamically, (2) the ensemble of low energy structures of control sequences deviate slightly more from the target pre-miRNA structure, as is the case for wild type pre-miRNA sequences, as mesured by ensemble defect [20], expected base pair distance to target [9], expected proportion of native contacts (called ensemble neutrality in [21]), average positional entropy [22], Morgan-Higgs structural diversity [23], and Vienna structural diversity (called *ensemble diversity* in [14]).

**Table 2:** Analysis of *C. elegans* precursor microRNA from the database miRBase 20 [11]

MEASURE	Def. Exact GC	Def. No GC	MFE Exact GC	MFE No GC	WT
BP DIST TARGET	0.06 ±0.03	0.09 ±0.04	0 ±0	0 ±0	0 ±0
ENERGY MFE	–0.53 ±0.11	–0.85 ±0.12	–0.48 ±0.12	–0.79 ±0.14	–0.38 ±0.11
ENERGY TARGET	–0.46 ±0.12	–0.78 ±0.14	–0.48 ±0.12	–0.79 ±0.14	–0.38 ±0.11
ENSEMBLE DEFECT	0.12 ±0.04	0.14 ±0.06	0.05 ±0.02	0.05 ±0.02	0.08 ±0.05
EXP BP DIST	0.07 ±0.03	0.1 ±0.04	0.03 ±0.01	0.03 ±0.01	0.05 ±0.03
PROP NAT CONTACT	0.93 ±0.04	0.9 ±0.06	0.96 ±0.02	0.96 ±0.02	0.92 ±0.05
POS ENTROPY	0.14 ±0.05	0.14 ±0.05	0.13 ±0.05	0.12 ±0.05	0.2 ±0.11
GC CONTENT	42.88 ±9.14	82.07 ±3.5	42.9 ±9.15	80.92 ±4.09	42.88 ±9.14
LN DUAL PROB	–95.4 ±21.03	–51.43 ±11.98	–102.73 ±22.81	–59.52 ±14.64	–117.11 ±25.94
LN PROB	-10.81 ±4.05	–10.95 ±4.68	–1.38 ±0.55	–0.96 ±0.45	–2.02 ±0.97
MH STR DIV	0.08 ±0.03	0.08 ±0.03	0.07 ±0.03	0.06 ±0.03	0.11 ±0.06
VIENNA STR DIV	0.05 ±0.02	0.05 ±0.02	0.04 ±0.02	0.04 ±0.02	0.07 ±0.04

For each of the 500 wild type (WT) pre-miRNA sequences, RNAdualPF sampled sequences, either having exactly the same GC-content as the WT sequence (‘Exact GC’) or with no control over GC-content (‘No GC’). The designation ‘MFE’ indicates that the sampled sequences were subsequently filtered to retain only those, whose minimum free energy structure is identical to the MFE structure of the corresponding WT pre-miRNA; otherwise, the designation ‘Def’ is used to indicate the default output of RNAdualPF, without the subsequent filtering step. For each WT pre-miRNA sequence, RNAdualPF generated 2000 sequences for the default case *Def* (no subsequent filtering), and 500 sequences for the non-default case *MFE*, such that sample MFE structure is identical to WT MFE structure. Various measures were used to compare the properties of RNAdualPF sampled sequences to those of wild type sequences: *BP DIST TARGET*: length-normalized average base pair distance *d*_*BP*_(*s*_0_,*s* ^∗^) between the MFE structure *s*_0_ of sequences sampled by RNAdualPF and the target structure *s* ^∗^. *ENERGY MFE*: length-normalized average free energy *E*(*s*_0_) of MFE structure *s*_0_. *ENERGY TARGET*: length-normalized average free energy *E*(*s* ^∗^) of target *s* ^∗^ for the respective sequences. *ENSEMBLE DEFECT*: length-normalized expected Hamming distance to target *s* ^∗^ [20]. *EXP BP DIST*: length-normalized expected base pair distance to target *s* ^∗^ [9]. *PROP NAT CONTACT*: expected proportion of base pairs of target *s* ^∗^ that occur in the MFE structure, i.e. $\langle \frac {|s_{0} \cap s^{*}|}{|s^{*}|} \rangle $. *POS ENTROPY*: average positional entropy [22]. *GC CONTENT*: average proportion of positions occupied by G or C. *LN DUAL PROB*: average natural logarithm of the dual probability exp(−*E*(**a,s**)/*RT*)/*Z*∗(**s**) that sequence **a** adopts the structure *s*. *LN PROB*: average natural logarithm of the probability exp(−*E*(**a,s**)/*RT*)/*Z*(**a**) that sequence **a** adopts the structure *s*. *MH STR DIV*: length-normalized Morgan-Higgs structural diversity [23]. *VIENNA STR DIV*: length-normalized Vienna structural diversity, called *ensemble diversity* in [14]. Values of all measures for default sampled sequences having GC-content within 5 % of wild type GC-content (not shown) are essential identical to those of exact GC-content control

Tables 3 and 4 display a similar analysis of the collection of bacterial small noncoding RNAs of [3] and of Rfam 12.0 database [24]. For the Rfam database, we selected one sequence from each of the ≈2500 Rfam families, with the property that the MFE structure of the sequence most resembled the Rfam consensus structure – i.e. whose MFE structure has smallest base pair distance to the consensus structure. These tables show similar trends as those displayed in Table 2, although values are larger due to increased sequence length of bacterial sncRNA and sequences from Rfam.

**Table 3:** Analysis of bacterial RNAs [3]

MEASURE	GC 5 %	Exact GC	No GC	WT
BP DIST TARGET	0.08 ±0.04	0.08 ±0.04	0.14 ±0.06	0 ±0
ENERGY MFE	–0.44 ±0.1	–0.44 ±0.1	–0.63 ±0.14	–0.29 ±0.1
ENERGY TARGET	–0.39 ±0.12	–0.39 ±0.12	–0.54 ±0.16	–0.29 ±0.1
ENSEMBLE DEFECT	0.16 ±0.08	0.16 ±0.08	0.23 ±0.1	0.14 ±0.09
EXP BP DIST	0.09 ±0.04	0.09 ±0.04	0.15 ±0.06	0.09 ±0.06
PROP NAT CONTACT	0.89 ±0.09	0.89 ±0.09	0.8 ±0.12	0.83 ±0.15
POS ENTROPY	0.19 ±0.08	0.19 ±0.08	0.23 ±0.09	0.35 ±0.18
GC CONTENT	48.33 ±7.02	48.34 ±7.02	74.75 ±5.55	48.34 ±7.02
LN DUAL PROB	–94.59 ±27.22	–94.54 ±27.19	–66.37 ±16.96	–117.22 ±33.37
LN PROB	–10.12 ±4.04	–10.09 ±4.03	–13.71 ±5.16	–2.34 ±0.95
MH STR DIV	0.1 ±0.04	0.1 ±0.04	0.13 ±0.05	0.18 ±0.09
VIENNA STR DIV	0.06 ±0.03	0.06 ±0.03	0.09 ±0.03	0.11 ±0.06

See Table 2 for an explanation of column headers and various measures. Since bacterial noncoding RNA is generally much longer than precursor microRNA, no subsequent filtering step was undertaken to ensure that sample sequence MFE structure is identical to that of wild type pre-miRNA. However an additional column is given for sequences required by RNAdualPF to have GC-content is within 5 % of WT value. (column header GC 5 %)

**Table 4:** Analysis of the Rfam 12.0 database

MEASURE	GC 5 %	Exact GC	No GC	WT
BP DIST TARGET	0.1 ±0.05	0.1 ±0.05	0.16 ±0.07	0 ±0
ENERGY MFE	–0.43 ±0.13	–0.43 ±0.13	–0.64 ±0.16	–0.28 ±0.13
ENERGY TARGET	–0.36 ±0.14	–0.36 ±0.14	–0.54 ±0.19	–0.28 ±0.13
ENSEMBLE DEFECT	0.18 ±0.07	0.18 ±0.07	0.25 ±0.11	0.16 ±0.12
EXP BP DIST	0.11 ±0.05	0.11 ±0.05	0.17 ±0.07	0.1 ±0.08
PROP NAT CONTACT	0.87 ±0.09	0.87 ±0.09	0.78 ±0.14	0.81 ±0.17
POS ENTROPY	0.22 ±0.09	0.21 ±0.09	0.25 ±0.1	0.4 ±0.25
GC CONTENT	46.27 ±10.91	46.27 ±10.91	75.12 ±5.89	46.27 ±10.91
LN DUAL PROB	–110.35 ±54.12	–110.34 ±54.12	–73.5 ±33.32	–136.7 ±65.62
LN PROB	–13.94 ±7.74	–13.9 ±7.71	–18.11 ±10.59	–2.83 ±1.71
MH STR DIV	0.12 ±0.05	0.12 ±0.05	0.13 ±0.05	0.2 ±0.12
VIENNA STR DIV	0.07 ±0.03	0.07 ±0.03	0.09 ±0.04	0.13 ±0.08

For each RNA family from Rfam 12.0, we selected that sequence whose MFE structure had smallest base pair distance to the Rfam consensus structure for the family. These sequences constituted the collection WT. See Table 3 for an explanation of column headers and various measures

**Fig. 3:**
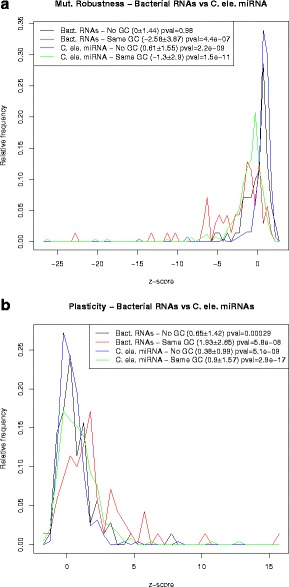
Z-scores of *mutational robustness* (**a**) and of *plasticity* (**b**) are presented for the bacterial small noncoding RNA (sncRNA) collection from [3] and for *C. elegans* precursor microRNA (pre-miRNA) from miRBase 20. For each wild type pre-miRNA [resp. sncRNA] wild type sequence, RNAdualPF sampled 2000 [resp. 1000] sequences using the minimum free energy structure of the wild type sequence as target structure. The GC-content of the sampled sequences was either required to be exactly that of the wild type sequence, or not (default mode of RNAdualPF), as indicated in the legend. Sampled sequences were used to compute the mutational robustness and plasticity, as explained in the main text. Note that *C. elegans* miRNA is significantly *robust* if GC-content is not controlled, but significantly *non-robust* if GC-content of RNAdualPF samples is identical to that of wild type pre-miRNA. Similarly, bacterial sncRNAs are not significantly robust if GC-content is not controlled, but significantly non-robust when GC-content is identical to that of wild type sncRNA. For this figure, mutational robustness of RNA sequence **a**=*a*_1_,…,*a*_*n*_ is defined by $1 - \frac {\langle D_{\text {\sc bp}}\rangle }{n}$, where ensemble distance *D*_bp_(**a**,**b**) between two length *n* sequences **a** and **b** is defined in [14], and the average ensemble distance from all single-point mutants of **a** is defined by $\langle D_{\text {\sc bp}}\rangle = \sum _{\mathbf {b}} \frac {D_{\text {\textsc {bp}}}(\mathbf {a},\mathbf {b})}{3n}$ where the sum is taken over all single-point mutants **b** of **a**. We use this notation of mutational robustness, rather than the notion defined in [3], since the latter notion is not a true metric, as explained in “Formal definitions of robustness” section. The plasticity $P = \frac {\langle D_{\text {\sc v}}\rangle }{n/2} = \sum _{i<j}\frac {p_{i,j}(1-p_{i,j})}{n}$ is defined in [3] as normalized *ensemble diversity*, where ensemble diversity [14] (Vienna structural diversity) *D*_V_ is defined by Eq. (4)

**Fig. 4:**
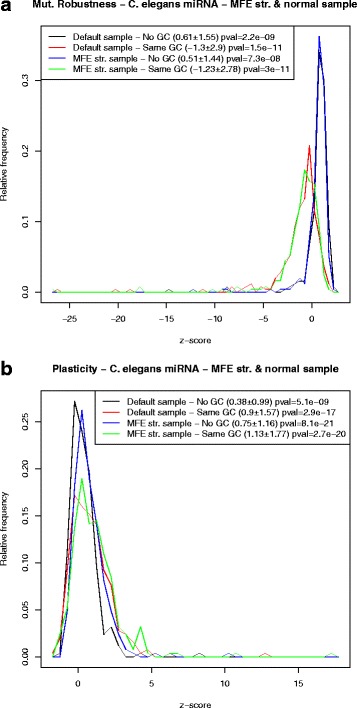
Z-scores of *mutational robustness* (**a**) and of *plasticity* (**b**) are presented for *C. elegans* microRNA from Rfam 12.0. Robustness and plasticity were measured as explained in Fig. 3. For each wild type (WT) sequence, 2000 sequences were sampled from RNAdualPF both with and without control over GC-content (default sample), while 500 sequences were generated by RNAdualPF, both with and without control over GC-content, to *exactly* fold into the target structure (MFE str. sample). This was achieved by repeatedly sampling sequences in order to obtain a sequence, whose MFE structure was identical to the target structure. The number of sequences necessary to sample in order to obtain one sequence that folds into the target structure was variable, depending on the target structure and GC-content – in many cases, only 10 samples were necessary per selected sequence, in some cases 200 samples were necessary. and in one specific case 5000 samples were required. By *control* over GC-content, we mean that all sampled sequences have identical GC-content to the wild type sequence

In agreement with [1], the left panel of Fig. 3 shows that *C. elegans* miRNA is significantly robust (Z-score of 0.61±1.55, 2-tailed T-test *p*-value 2.2×10^−9^), *provided* that GC-content is *not* controlled. However, in contrast to [1], when GC-content is controlled, we find that *C. elegans* miRNA is significantly *non-robust* (Z-score of −1.3±2.9, 2-tailed T-test *p*-value 1.5×10^−11^). To corroborate our findings, for each wild type *C. elegans* pre-miRNA, we performed a second computational experiment, to generate 500 sequences with no control over GC-content and 500 sequences whose GC-content was identical with that of the wild type pre-miRNA. In contrast to the first experiment, we used RNAdualPF to generate sufficiently many sequences to subsequently select 500 sequences (no GC-control) and 500 sequences (GC-content equal to wild type pre-miRNA), each of whose MFE structure was identical to that of wild type pre-miRNA. The left panel of Fig. 4 shows that when GC-content is not controlled, *C. elegans* precursor microRNAs are statistically robust (Z-score 0.51±1.44, *p*-value 7.3×10^−8^), in agreement with the main result of Borenstein and Ruppin [1]. However, when GC-content of control sequences is identical to that of wild type precursor microRNA, we confirm that *C. elegans* pre-miRNA is *statistically non-robust* (Z-score −1.23±2.78, *p*-value 3×10^−11^). Note that our finding, which is in opposition to results of Borenstein and Ruppin [1], is based on a larger data set of precursor microRNAs, each of which has a larger control set, than in the analysis of [1].

Turning now to the analysis of bacterial small noncoding RNAs, we find that sncRNAs are not significantly robust (Z-score 0.0±1.4, *p*-value 0.98) when GC-content is *not controlled*, confirming a result from Rodrigo et al. [3]. However, when GC-content of the sequences sampled from RNAdualPF is required to be identical to that of wild type sncRNA, bacterial sncRNAs are see to be *significantly non-robust* (Z-score −2.58±3.87, *p*-value of 4.4×10^−7^). Note here that Rodrigo et al. used RNAinverse in their computational experiments, hence could not consider the case with control over GC-content. The left panels of Figs. 3 a and 4 summarize our findings that precursor microRNAs [resp. bacterial sncRNAs] are significantly non-robust [resp. not significantly robust] with respect to a control set of 2000 [resp. 1000] sequences generated by RNAdualPF with identical GC-content to that of the wild type sequence.

Finally, in our analysis of *plasticity*, the right panels of Figs. 3 and 4 show that both *C. elegans* and bacterial small noncoding RNAs exhibit more plasticity when compared with control sequences for which GC-content is not controlled, as well as when compared with control sequences for which GC-content is identical to that of wild type sequences.

### Structural RNA has higher free energy than expected

In Figure 4 of [9], we showed that the free energy *E*_0_ of the minimum free energy (MFE) structure *s*_0_ of *E. coli* val-tRNA (accession RV1600 from Sprinzl database [25] tdbR00000454 from tRNAdb [26]), is much *higher* (less favorable) than the average free energy 〈*E*〉 of over four million RNAs having the same MFE structure *s*_0_ as that of *E. coli* val-tRNA. Here, *E. coli* val-tRNA RV1600 was selected, because its MFE structure *s*_0_ is identical to the Rfam consensus structure for tRNA family RF00005. This preliminary result suggests that naturally occurring transfer RNAs may be under selective pressure to be only marginally thermodynamically stable. Since it took a number of days for RNAiFold [4, 9] to return over four million solutions of the inverse folding problem for the tRNA target structure, we now describe how RNAdualPF can be used to compute the Boltzmann expected free energy of literally all sequences *a*_1_,…,*a*_*n*_ with respect to an arbitrary target structure *s*_0_. In this manner, we confirm our preliminary finding concerning *E. coli* val-tRNA, and show that the folding energy of structural RNA from the Rfam database is much higher (less favorable) than expected. Before presenting results, we need some definitions.

For the Turner nearest neighbor energy model [13], the free energy of a secondary structure *s* of an RNA sequence **a**=*a*_1_,…,*a*_*n*_ depends on the (absolute) temperature *T*_0_. To indicate this dependence, we write *E*(**a,s,T**_0_), where in the sequel, *T*_0_ will be designated as *table temperature*, i.e. the temperature for which parameters from the Turner energy tables are applied. For an arbitrary, but fixed secondary structure *s*_0_ of length *n*, the *dual partition function* at temperature *T*_0_ is defined by

52 $$\begin{array}{*{20}l}  Z(s_{0},T_{0},T) = \sum_{\mathbf{a}} \exp\left(-E\left(\mathbf{a},s_{0},T_{0}\right)/RT\right) \end{array} $$

where the sum is taken over all RNA sequences **a**=*a*_1_,…,*a*_*n*_ of length *n*. Note that *T*_0_ indicates the (table) temperature at which the energy of a structure *s*_0_ and nucleotide sequence **a** is evaluated using the Turner parameters, while all other occurrences of the temperature variable are designated by *T*, which we call *formal temperature*. The distinction between formal and table temperature is made to allow us to use finite difference approximations to derivatives with respect to the *formal temperature* when when we compute *dual expected energy* and *dual conformational entropy* below (see [27] for more explanation). When table temperature *T*_0_ equals formal temperature *T*, and the temperature is clear from the context, we write *Z* ^∗^(*s*_0_); if the target structure *s*_0_ is also clear from the context, then we write *Z* ^∗^. A similar remark applies to the other thermodynamic functions *p* ^∗^,*G* ^∗^,〈*E* ^∗^〉,*S* ^∗^, which we now define.

The *dual Boltzmann probability* *p* ^∗^(**a**) is defined by

53 $$\begin{array}{*{20}l}  p^{*}\left(\mathbf{a},s_{0},T_{0},T\right) &= \frac{\exp\left(-E\left(\mathbf{a},s_{0},T_{0}\right)\right)}{Z^{*}\left(s_{0},T_{0},T\right)} \end{array} $$

The *dual ensemble free energy* *G* ^∗^(*s*_0_) is defined by

54 $$\begin{array}{*{20}l}  G^{*} &= G^{*}(s_{0}) = G\left(s_{0},T_{0},T\right) = -RT \ln Z^{*}\left(s_{0},T_{0},T\right) \end{array} $$

where *R*≈1.987 cal/(mol K) is the universal gas constant. The *dual expected (free) energy* 〈*E* ^∗^(*s*_0_)〉 is defined by

55 $$\begin{array}{*{20}l}  \langle E^{*}\left(s_{0},T_{0},T\right) \rangle &= \sum_{\mathbf{a}} E\left(\mathbf{a},s_{0},T_{0}\right) \cdot p\left(\mathbf{a},s_{0},T_{0},T\right) \end{array} $$

Straightforward derivations analogous to those in [27] yield the following expressions for *dual expected energy* 〈*E* ^∗^〉 and *dual entropy* *S* ^∗^:

56 $$\begin{array}{*{20}l}  \langle E^{*}\left(s_{0},T_{0},T\right) \rangle &= RT^{2} \cdot \frac{\partial}{\partial T} \left(\ln Z^{*}(s_{0},T_{0},T) \right)_{T=T_{0}} \end{array} $$

57 $$\begin{array}{*{20}l}  S^{*}\left(s_{0},T_{0},T\right) &= \frac{\langle E\left(\mathbf{a},s_{0},T_{0},T\right) \rangle - G^{*}\left(s_{0},T_{0},T\right)}{T} \end{array} $$

Programs to compute *dual expected energy* 〈*E* ^∗^〉, *dual conformational entropy* *S* ^∗^, and *dual heat capacity* $C^{*}_{p}$ are provided at our web site. We do not elaborate further on dual entropy or dual heat capacity, since at the present time we have found no compelling applications.

**Fig. 5:**
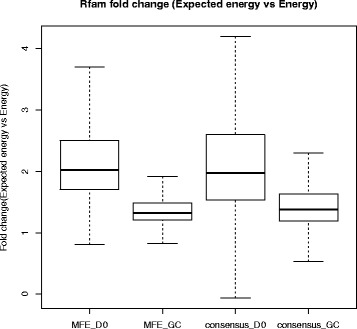
Analysis of expected free energy 〈*E*〉 for structures in Rfam 12.0 [24]. Given a secondary structure *s*, the expected free energy of all sequences **a** with respect to *s* is defined by $\langle E(s) \rangle = \sum _{\mathbf {a}} E(\mathbf {a},s) \cdot \frac {\exp (-E(\mathbf {a},s)/RT)}{Z^{*}(\mathbf {a},s)}$, where *Z* ^∗^ is the *dual partition function* defined in equation (7). For each Rfam family, we took the family consensus structure *s*_*c*_, and computed 〈*E*(*s*_*c*_)〉. Additionally, for each Rfam family, we selected that sequence **a**_0_, whose minimum free energy (MFE) structure *s*_0_ has smallest base pair distance to the consensus structure *s*_*c*_. The expected energy 〈*E*(*s*_0_)〉 was computed, as well as the free energies *E*(**a,s**_*c*_) and *E*(**a,s**_0_). The fold change $\frac {\langle E(s_{c}) \rangle }{E(\mathbf {a}_{0},s_{c})}$ for the consensus structure and the fold change $\frac {\langle E(s_{0}) \rangle }{E(\mathbf {a}_{0},s_{0})}$ for the minimum free energy structure were computed. The box-and-whiskers plots show the mean, 25th and 75th percentile, minimum and maximum values. As indicated in the legend, these computations were performed either with respect to all sequences or with respect to all sequences having the same (exact) GC-content. These data clearly indicate that natural RNA sequences, whose MFE structures most closely resemble the Rfam consensus structures, have *higher* free energy than expected

Figure 5 shows that structural RNAs have *higher* free energy with respect to their native structure, hence are thermodynamically *less stable*, than expected, – even when expectations are taken over all sequences having the same GC-content as that of wild type sequences. We believe that this insight could be important when designing functional synthetic RNAs. To generate Fig. 5, we proceeded as follows. For each family from the Rfam 12.0 database [24], we took the family consensus structure *s*_*c*_, and computed 〈*E*(*s*_*c*_)〉. Additionally, for each Rfam family, we selected that sequence **a**_0_, whose minimum free energy (MFE) structure *s*_0_ has smallest base pair distance to the consensus structure *s*_*c*_. We computed the expected energy 〈*E*(*s*_0_)〉, as well as the free energies *E*(**a,s**_*c*_) and *E*(**a,s**_0_). Figure 5 displays box-and-whiskers plots for the fold change $\frac {\langle E(s_{c}) \rangle }{E(\mathbf {a}_{0},s_{c})}$ for the consensus structure and the fold change $\frac {\langle E(s_{0}) \rangle }{E(\mathbf {a}_{0},s_{0})}$ for the minimum free energy structure. Since the dual Boltzmann probability *p* ^∗^(**a,s**_0_) is generally larger for sequences **a** having higher GC-content (as stacked base pairs involving GC,CG have lower free energy than those involving AU,UA,GU,UG), RNAdualPF computes as well the *dual partition function* for GC-content *k*, defined by

58 $$  Z^{\ast}(s_{0},k) = \sum_{\substack{\text{\textbf{a} such that}\\ \text{GC-content}=k}} \exp(-E(\mathbf{a},s_{0})/RT)  $$

In this fashion, we can exactly compute the *dual expected energy* 〈*E* ^∗^(*s*_0_,*k*)〉 of all sequences having GC-content *k* which approximately fold into target structure *s*_0_. Tables 2, 3 and 4 analyze what we mean by *approximately* folding into the target structure – i.e. sequences **a** are preferentially sampled when free energy *E*(**a,s**_0_) is low, hence have large dual Boltzmann probability. RNAdualPF, even when exact GC-content is controlled, is faster than inverse folding programs by orders of magnitude, hence providing an effective alternative manner of solving inverse folding.

## Conclusion

In this paper we describe the algorithm and software RNAdualPF, which computes the *dual partition function* *Z* ^∗^, defined as the sum of Boltzmann factors exp(−*E*(**a,s**_0_)/*RT*) of all *sequences* **a** with respect to the target structure *s*_0_. Using RNAdualPF, we efficiently sample RNA sequences that (approximately) fold into *s*_0_, where additionally the user can specify IUPAC sequence constraints at certain positions, and whether to include dangles (energy terms for stacked, single-stranded nucleotides). Moreover, the user can require that all sampled sequences have a precisely specified GC-content, since, optionally, we compute the *dual partition function* *Z* ^∗^(*k*) simultaneously for all values *k*=*G*+*C*. This sampling strategy is complementary to the use of RNAiFold [4], since it allows the study of the properties of long RNA structures whose number of solutions for the inverse folding problem is astronomically large.

We use RNAdualPF to corroborate previous studies [1] using RNAinverse [2], by confirming that precursor microRNAs are significantly mutationally robust when GC-content is not controlled. However, in contrast to [1], we find that precursor microRNAs are significantly *non-robust* when GC-content is controlled. We confirm and extend previous findings [3] that bacterial small noncoding RNAs display plasticity (structural diversity) and are not statistically robust, when GC-content is not controlled. Additionally, we obtain the new finding that when when GC-content is controlled, bacterial small noncoding RNAs are significantly non-robust, as in the case of precursor microRNAs. One possible reason for the discrepancy between our results and those of [1] could be related with the fact that the energy parameters of Vienna RNA Package 1.4 (Turner 1999 parameters used in the computational experiments of [1]) differ from those of Vienna RNA Package 2.1.9 (Turner 2004 parameters used in the current study with RNAdualPF). Another possible reason is that the inverse folding solutions returned by the program RNAinverse used in [1] show a different bias than sequences returned by RNAdualPF (in this context, we mean the inverse folding solutions filtered from the sequences returned by RNAdualPF).

As mentioned in the Introduction, there is a relation between our C program RNAdualPF and the Python program IncaRNAtion [12], although our work is independent of that of Reinharz et al. [12]. IncaRNAtion is a weighted sampling algorithm that computes the dual partition function for a simple energy model, which only considers base stacking free energies – unlike RNAdualPF, the program IncaRNAtion includes no energy contributions for hairpins, bulges, internal loops, multiloops, dangles, or mismatches. If the user specifies a desired GC-content *α*, then IncaRNAtion does not compute the dual partition function for GC-content, but rather applies an adjustable heuristic so that after a suitable *burn-in* period, sequences tend to approximately have GC-content *α*. See Table 6.2 of [28] for benchmarking results on RNAdualPF and IncaRNAtion, which show conclusively that RNAdualPF is not only faster, but its sequences have a higher probability of folding into the target structure, its sequences have a smaller GC-content in default mode, where GC-content is not controlled, etc.

**Fig. 6:**
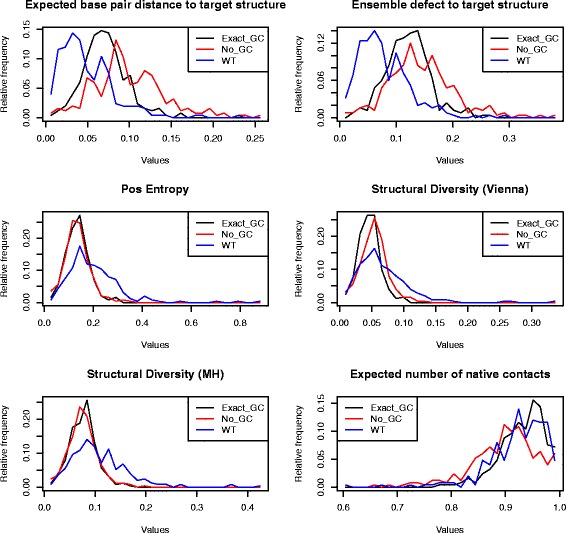
For each of the 250 *C. elegans* precursor microRNAs from miRBase 20 and for each of the following cases (**a**), indicated in *black*, and (**b**), indicated in *red*, RNAdualPF sampled 2000 sequences without any subsequent filtering step. Case (**a**) - *black* lines: All RNAdualPF sequences have GC-content exactly equal to that of the Rfam sequence (Exact GC). Case (**b**) - *red* lines: RNAdualPF was used in default mode, without controlling GC-content (No GC). Case (**c**) - *blue* lines: wild type (WT) *C. elegans* data. Density plots are shown for (1) the expected base pair distance to target structure *s*_0_ [9], (2) the ensemble defect to target structure *s*_0_ [20], (3) the positional entropy [22], (4) Vienna structural diversity (called ensemble diversity in [14]), (5) Morgan-Higgs diversity [23], (6) expected proportion of native contacts (called ensemble neutrality in [21]). All measures were normalized by sequence length

**Fig. 7:**
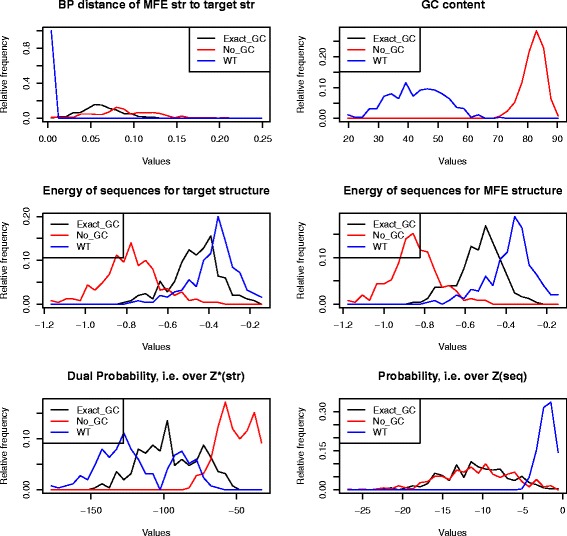
Additional measures for the data described in the previous Fig. 6. Density plots are shown for (1) the base pair distance between the minimum free energy (MFE) structure and the target structure, (2) the GC-content, (3) the free energy *E*(**a,s**_0_) of the RNA sequences **a** with respect to the target structure *s*_0_, (4) the free energy *E*(**a,s**_*a*_) of each sequence with respect to its own minimum free energy (MFE) structure, (5) the log dual probability $p^{*}(s_{0}) = \frac {\sum _{\mathbf {x}} \exp (-E(\mathbf {x},s_{0})/RT)}{Z^{*}(s_{0})}$, and (6) the log probability $p(\mathbf {a}) = \frac {\sum _{s} \exp (-E(\mathbf {a},s)/RT)}{Z(\mathbf {a})}$

**Fig. 8:**
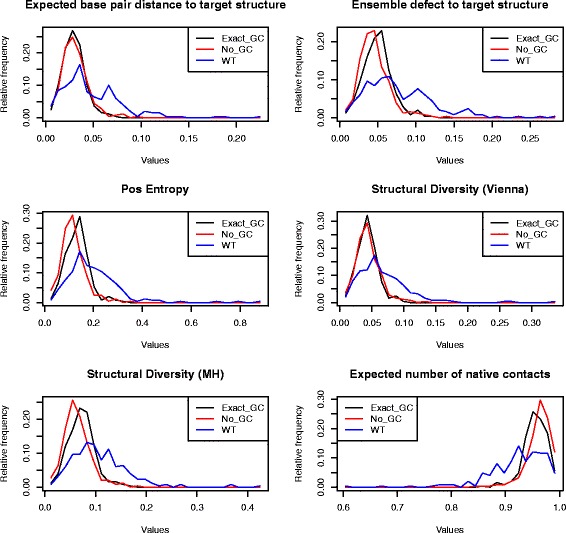
For each of the 250 *C. elegans* precursor microRNAs from miRBase 20 and for each of the following cases (**a**), indicated in *black*, and (**b**), indicated in *red*, RNAdualPF generated 500 sequences, whose minimum free energy structure was *identical* to that of the corresponding wild type pre-miRNA (obtained by repeatedly generating samples with RNAdualPF until 500 sequences were found that folded exactly into the target structure). Case (**a**) - *black* lines: All RNAdualPF sequences have GC-content exactly equal to that of the Rfam sequence (Exact GC). Case (**b**) - red lines: RNAdualPF was used in default mode, without controlling GC-content (No GC). Case (**c**) - *blue* lines: wild type (WT) *C. elegans* data. Density plots are shown for (1) the expected base pair distance to target structure *s*_0_ [9], (2) the ensemble defect to target structure *s*_0_ [20], (3) the positional entropy [22], (4) Vienna structural diversity (called ensemble diversity in [14]), (5) Morgan-Higgs diversity [23], (6) expected proportion of native contacts (called ensemble neutrality in [21]). All measures were normalized by sequence length

**Fig. 9:**
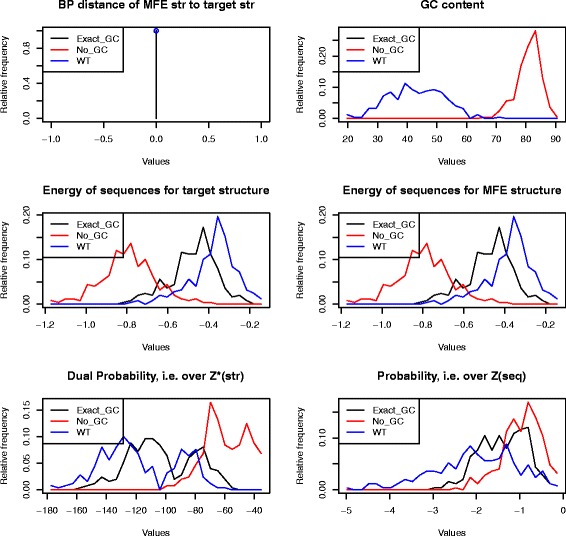
Additional measures for the data described in the previous Fig. 8. Density plots are shown for (1) the base pair distance between the minimum free energy (MFE) structure and the target structure, (2) the GC-content, (3) the free energy *E*(**a,s**_0_) of the RNA sequences **a** with respect to the target structure *s*_0_, (4) the free energy *E*(**a,s**_*a*_) of each sequence with respect to its own minimum free energy (MFE) structure, (5) the log dual probability $p^{*}(s_{0}) = \frac {\sum _{\mathbf {x}} \exp (-E(\mathbf {x},s_{0})/RT)}{Z^{*}(s_{0})}$, and (6) the log probability $p(\mathbf {a}) = \frac {\sum _{s} \exp (-E(\mathbf {a},s)/RT)}{Z(\mathbf {a})}$

Our original motivation in designing RNAdualPF was to generate an *unbiased* sample of *near-solutions* (or by subsequent selection of *solutions*) to the inverse folding problem. At present, it seems clear that no program can claim to generate an unbiased sample of inverse folding solutions, since (1) the solution space so large that this hypothesis cannot be tested by brute force methods, and (2) different inverse folding algorithms return solution sequences having different properties, as shown in Table 2 of [4]. Nevertheless, in the same manner that structures sampled by the algorithm of Ding and Lawrence [16] constitute an unbiased, representative set of low energy secondary structures for a given RNA sequence, as implemented in Sfold [10] and RNAsubopt -p [2], the collection of RNA sequences sampled by the algorithm RNAdualPF constitute an unbiased, representative set of sequences having low energy with respect to a given target structure *s*_0_. Although the minimum free energy structure of such sequences may indeed be distinct from *s*_0_, it is likely that the MFE structure and the target *s*_0_ be similar, as shown in Tables 2, 3 and 4. Moreover, Fig. 6 presents relative frequency plots that suggest that when GC-content is controlled, the sequences returned by RNAdualPF have similar properties to those of wild type sequences: (1) similar expected base pair distance to the wild type target structure [9], (2) similar ensemble defect to the target wild type structure [20], (3) similar positional entropy [22], (4) similar Vienna structural diversity (called ensemble diversity in [14]), (5) similar Morgan-Higgs diversity [23], (6) similar expected proportion of native contacts (called ensemble neutrality in [21]). These graphs were produced by using RNAdualPF to sample 2,000 sequences for each of the 250 *C. elegans* precursor microRNAs from the miRBase 20 database [11], in each of the following cases: (a) GC-content identical to that of the Rfam sequence, (b) no control for the GC-content. Figure 7 presents additional data, computed in the same manner for *C. elegans* pre-miRNA from miRBase 20, showing that when GC-content is controlled, sequences sampled by RNAdualPF satisfy the following: (1) the average length-normalized base pair distance between the minimum free energy and target structures is ≈0.05, (2) wild type and RNAdualPF sampled sequences have similar free energy with respect to the wild type target structure, (3) as well as similar minimum free energy, (4) similar dual probability, and (5) similar probability to wild type RNA sequences. Taken together, this data shows that if GC-content is controlled, then RNAdualPF returns sequences whose low energy structures tend to resemble the target structure. Figures 8 and 9 are similar to Figs. 6 and 7, except that for each *C. elegans* pre-miRNA, 500 sequences were generated by RNAdualPF, each of whose MFE structure is *identical* to the wild type target structure (this was done by repeatedly sampling sequences from RNAdualPF until 500 sequences were found, that fold exactly into the target pre-miRNA structure). Taken together, Figs. 6, 7, 8 and 9 present convincing evidence that RNAdualPF generates sequences that (approximately) fold into the user-specified target structure, hence supporting our finding that *C. elegans* precursor microRNAs are statistically non-robust, contrary to the finding of [1].

Additionally, we have shown that natural RNAs from the Rfam 12.0 database have *higher* minimum free energy than expected, thus supporting our results in [9] which suggest that functional RNAs are under evolutionary pressure to be only marginally thermodynamically stable. The applications described in this paper demonstrate that RNAdualPF is a useful and extremely fast software tool for evolutionary and synthetic biology.

## Endnote

^1^ When dangling positions are not included in the computation (-d0), the algorithm clearly requires linear time. When dangling positions are included (-d2), run time is exponential in the number of components of the largest multilooop; however, in practice the algorithm is extremely fast, and it is possible to modify the algorithm to always run in linear time.

## Additional file

Additional file 1 Supplementary Information. Title is *Supplementary Information for RNAdualPF: software to compute the dual partition function with sample applications in molecular evolution theory*. This is a 3-page PDF file containing a tricky derivation for an efficient computation of the number of external loops of size *N* with GC-content *k*, where the user can stipulate that certain positions are constrained to contain nucleotides consistent with IUPAC codes. (PDF 273 kb)

## Abbreviations

MFE: Minimum free energy
PLMVd: Peach latent mosaic viroid
sncRNA: Small noncoding RNA

## References

[1] Borenstein E, Ruppin E (2006). Direct evolution of genetic robustness in microRNA. Proc Natl Acad Sci.

[2] Lorenz R, Bernhart SH, Honer Zu Siederdissen C, Tafer H, Flamm C, Stadler PF, Hofacker IL (2011). ViennaRNA Package 2.0. Algorithms Mol Biol.

[3] Rodrigo G, Fares MA (2012). Describing the structural robustness landscape of bacterial small RNAs. BMC Evol Biol.

[4] Garcia-Martin JA, Dotu I, Clote P (2015). RNAiFold 2.0: a web server and software to design custom and rfam-based RNA molecules. Nucleic Acids Res.

[5] Los Alamos HIV database. 2015. http://www.hiv.lanl.gov/. Accessed 30 Dec 2015.

[6] Krol J, Sobczak K, Wilczynska U, Drath M, Jasinska A, Kaczynska D, Krzyzosiak WJ (2004). Structural features of microRNA (miRNA) precursors and their relevance to mirna biogenesis and small interfering RNA/short hairpin RNA design. J Biol Chem.

[7] Hofacker IL, Fontana W, Stadler PF, Bonhoeffer LS, Tacker M, Schuster P (1994). Fast folding and comparison of RNA secondary structures. Monatsch Chem.

[8] Zadeh JN, Wolfe BR, Pierce NA (2011). Nucleic acid sequence design via efficient ensemble defect optimization. J Comput Chem.

[9] Garcia-Martin JA, Clote P, Dotu I (2013). RNAiFold: a constraint programming algorithm for RNA inverse folding and molecular design. J Bioinform Comput Biol.

[10] Ding Y, Chan CY, Lawrence CE (2004). Sfold web server for statistical folding and rational design of nucleic acids. Nucleic Acids Res.

[11] Kozomara A, Griffiths-Jones S (2014). mirbase: annotating high confidence microRNAs using deep sequencing data. Nucleic Acids Res.

[12] Reinharz V, Ponty Y, Waldispuhl J (2013). A weighted sampling algorithm for the design of RNA sequences with targeted secondary structure and nucleotide distribution. Bioinformatics.

[13] Turner DH, Mathews DH (2010). NNDB: the nearest neighbor parameter database for predicting stability of nucleic acid secondary structure. Nucleic Acids Res.

[14] Gruber AR, Bernhart SH, Hofacker IL, Washietl S (2008). Strategies for measuring evolutionary conservation of RNA secondary structures. BMC Bioinforma.

[15] McCaskill JS (1990). The equilibrium partition function and base pair binding probabilities for RNA secondary structure. Biopolymers.

[16] Ding Y, Lawrence CE (2003). A statistical sampling algorithm for RNA secondary structure prediction. Nucleic Acids Res.

[17] Busch A, Backofen R (2006). INFO-RNA, a fast approach to inverse RNA folding. Bioinformatics.

[18] Zuker M, Mathews DH, Turner DH, Barciszewski J, Clark BFC (1999). Algorithms and thermodynamics for RNA secondary structure prediction: A practical guide. RNA Biochemistry and Biotechnology. NATO ASI Series.

[19] Griffiths-Jones S, Bateman A, Marshall M, Khanna A, Eddy SR (2003). Rfam: an RNA family database. Nucleic Acids Res.

[20] Dirks RM, Lin M, Winfree E, Pierce NA (2004). Paradigms for computational nucleic acid design. Nucleic Acids Res.

[21] Pei S, Anthony JS, Meyer MM (2015). Sampled ensemble neutrality as a feature to classify potential structured RNAs. BMC Genomics.

[22] Huynen M, Gutell R, Konings D (1997). Assessing the reliability of RNA folding using statistical mechanics. J Mol Biol.

[23] Morgan SR, Higgs PG (1998). Barrier heights between ground states in a model of RNA secondary structure. J Phys A: Math Gen.

[24] Nawrocki EP, Burge SW, Bateman A, Daub J, Eberhardt RY, Eddy SR, Floden EW, Gardner PP, Jones TA, Tate J, Finn RD (2015). Rfam 12.0: updates to the RNA families database. Nucleic Acids Res.

[25] Sprinzl M, Horn C, Brown M, Ioudovitch A, Steinberg S (1998). Compilation of tRNA sequences and sequences of tRNA genes. Nucleic Acids Res.

[26] Juhling F, Morl M, Hartmann RK, Sprinzl M, Stadler PF, Putz J (2009). tRNAdb 2009: compilation of tRNA sequences and tRNA genes. Nucleic Acids Res.

[27] Garcia-Martin JA, Clote P (2015). RNA Thermodynamic Structural Entropy. PLoS ONE.

[28] Garcia-Martin JA. RNA inverse folding and synthetic design. Ph.D. dissertation in Biology, Boston College. 2016. Dissertation made available on June 28, 2016 and will remain accessible indefinitely: http://hdl.handle.net/2345/bc-ir:106989.

