# Supplementary material for: RNAdualPF: software to compute the dual partition function with sample applications in molecular evolution theory
Source: BMC Bioinformatics. 2016 Oct 19;17:424. doi: 10.1186/s12859-016-1280-6 (PMC5069997; doi:10.1186/s12859-016-1280-6)
Supplement: Additional file 1 — Supplementary Information. Title is Supplementary Information for RNAdualPF: software to compute the dual partition function with sample applications in molecular evolution theory. This is a 3-page PDF file containing a tricky derivation for an efficient computation of the number of external loops of size N with GC-content k, where the user can stipulate that certain positions are constrained to contain nucleotides consistent with IUPAC codes. (PDF 273 kb) [file 12859_2016_1280_MOESM1_ESM.pdf]

Supplementary Information
RNAdualPF: software to compute the dual partition function with sample applications in molecular evolution theory

J.A. Garcia-Martin[1], A.H. Bayegan[1], I. Dotu[2], P. Clote[1,*]

1: Department of Biology, Boston College, Chestnut Hill, MA 02467 USA.
2: Research Programme on Biomedical Informatics (GRIB), Department of Experimental and Health Sciences, Universitat Pompeu Fabra, IMIM (Hospital del Mar Medical Research Institute), Dr. Aiguader 88, Barcelona, Spain.

Here we describe a simple, yet tricky, combinatorial algorithm to efficiently count the number N of external loops of size n with GC-content k, where the user can stipulate that certain positions are constrained to contain nucleotides consistent with IUPAC codes. If there are no IUPAC constraints, then clearly $N = \binom{n}{k} \cdot 2^k \cdot 2^{n-k} = \binom{n}{k} \cdot 2^n$; however, with IUPAC constraints for uncertain data, the situation is a good deal more complicated. In order to explain and justify the algorithm, we introduce some definitions which may appear pedantic at this point, but most certainly are not and will simplify presentation of the algorithm.

Class $A = \{R, Y, M, K\}$ is defined to be the set consisting of IUPAC codes R,Y,M,K, where R is A or G, Y is C or U, M is A or C, K is G or U. Note that if m nucleotide positions are constrained by an IUPAC code belonging to class A, and k of these positions are required to have GC-content of k, then the number of sequences satisfying this requirement is $\binom{m}{k} \cdot 1^k \cdot 1^{m-k} = \binom{m}{k}$.

Class $B = \{B, V\}$ is defined to be the set consisting of IUPAC codes B,V where B is C or G or U (i.e. not A), V is A or C or G (i.e. not U). Note that if m nucleotide positions are constrained by an IUPAC code belonging to class B, and k of these positions are required to have GC-content of k, then the number of sequences satisfying this requirement is $\binom{m}{k} \cdot 2^k \cdot 1^{m-k} = \binom{m}{k} \cdot 2^k$.

Class $C = \{D, H\}$ is defined to be the set consisting of IUPAC codes D,H, where D is A or G or U (i.e. not C), H is A or C or U (i.e. not G). Note that if m nucleotide positions are constrained by an IUPAC code belonging to class C, and k of these positions are required to have GC-content of k, then the number of sequences satisfying this requirement is $\binom{m}{k} \cdot 1^k \cdot 2^{m-k} = \binom{m}{k} \cdot 2^{m-k}$.

Class $D = \{N\}$ is defined to be the set consisting of IUPAC code N, where N is A or C or G or U (i.e. any nucleotide). Note that if m nucleotide positions are constrained by an IUPAC code belonging to class D, and k of these positions are required to have GC-content of k, then the number of sequences satisfying this requirement is $\binom{m}{k} \cdot 2^k \cdot 2^{m-k} = \binom{m}{k} \cdot 2^m$.

Class $E = \{S\}$ is defined to be the set consisting of IUPAC code S, where S is G or C (i.e. strong). Note that if m nucleotide positions are constrained by an IUPAC code belonging to class E, then there are 2^m many such sequences satisfying this constraint.

Class $F = \{W\}$ is defined to be the set consisting of IUPAC code W, where W is A or U (i.e. weak). Note that if m nucleotide positions are constrained by an IUPAC code belonging to class F, then there are 2^m many such sequences satisfying this constraint.

Class $G = \{A, C, G, U\}$ is defined to be the set consisting of IUPAC codes A,C,G,U (i.e. the data is certain).

*Corresponding author P. Clote. Email addresses j.antonio.garciamartin@gmail.com, a.h.bayegan@gmail.com, ivan.dotu@gmail.com, clote@bc.edu

Note that there are 15 IUPAC codes, of which 11 concern *uncertain* data; indeed, only IUPAC codes in class G concern *certain* data.

Algorithm 1 (Number of external loops of size n having GC-content of k, allowing IUPAC constraints)

Input: *Integer $n \geq 1$ denoting the length of the external loop, integer $k \geq 0$ denoting the desired GC-content of the external loop, length n sequence of IUPAC constraints specified by $\mathbf{a} = a_1, \ldots, a_n$; i.e. for each $i = 1, \ldots, n$, we have $a_i \in \{A, C, G, U, R, Y, M, K, B, V, D, H, N, S, W\}$.*
Output: *Number of external loops of size n having GC-content of k, which satisfy the specified IUPAC constraints.*

Define the following.

- Let $n_a, n_b, n_c, n_d, n_e, n_f, n_g$ denote the number of positions in the external loop that are constrained by an IUPAC code belonging respectively to class A, B, C, D, E, F, G.
- Let $n_0 = n - (n_e + n_f + n_g)$. Note that n_0 is the number of positions in the external loop that may be assigned to contain G or C, or equally well may be assigned to contain A or U. We must have that $n_0 = n_a + n_b + n_c + n_d$, hence $n_d = n_0 - (n_a + n_b + n_c)$.
- Let num_C [resp. num_G] denote the number of positions in the external loop that are constrained by IUPAC code C [resp. G]. Note that a position constrained to be C [resp. G] will contribute both to the count of n_g as well as to the count of num_C [resp. num_G].
- Let $k_0 = k - (num_C + num_G + n_e)$. Note that k_0 is the number of C's or G's that must be assigned among the n_0 positions, taking into consideration that we have already taken care of assignments of C's and G's to positions that are constrained to be C (there are num_C many), or G (there are num_G many), or either C or G (there are n_e many).
- Let k_a, k_b, k_c, k_d denote the number of positions constrained by IUPAC codes that belong respectively to class A, B, C, D that will be set to contain either C or G. Although k_a, k_b, k_c, k_d will take on different values, we will always ensure that $k_0 = k_a + k_b + k_c + k_d$, hence $k = k_a + k_b + k_c + k_d + n_e + num_C + num_G$; in particular, $k_d = k_0 - (k_a + k_b + k_c)$. As well, it clearly must always hold that $0 \leq k_a \leq n_a$, $0 \leq k_b \leq n_b$, $0 \leq k_c \leq n_c$, $0 \leq k_d \leq n_d$.

Careful scrutiny justifies the fact that the number N of external loops of size n having GC-content of k, which satisfy the specified IUPAC constraints, must satisfy the following.

$$N = \sum_{k_a=0}^{n_a} \sum_{k_b=0}^{n_b} \sum_{k_c=0}^{n_c} \binom{n_a}{k_a} \cdot \binom{n_b}{k_b} \cdot \binom{n_c}{k_c} \cdot \binom{n_d}{k_d} \cdot \tag{1}$$

$$(1^{k_a} \cdot 1^{n_a - k_a}) \cdot (2^{k_b} \cdot 1^{n_b - k_b}) \cdot (1^{k_c} \cdot 2^{n_c - k_c}) \cdot 2^{n_e} \cdot 2^{n_f} \cdot$$
$$2^{k_0 - (k_a + k_b + k_c)} \cdot 2^{n_d - (k_0 - (k_a + k_b + k_c))} \tag{2}$$
$$= \sum_{k_a=0}^{n_a} \sum_{k_b=0}^{n_b} \sum_{k_c=0}^{n_c} \binom{n_a}{k_a} \cdot \binom{n_b}{k_b} \cdot \binom{n_c}{k_c} \cdot \binom{n_d}{k_d} \cdot 2^{n_c + n_d + n_e + n_f} \cdot 2^{k_b - k_c}$$

This leads to the following pseudocode.

```
def f(a) //compute n_a, n_b, n_c, n_d, n_e, n_f, n_g, num_C, num_G from IUPAC constraints
//a = a_1, ..., a_n stipulates IUPAC constraints at all positions of external loop
    n_a = n_b = n_c = n_d = n_e = n_f = n_g = num_C = num_G = 0
    for i = 1 to n
        if a_i ∈ {R, Y, M, K}
```

n_a += 1

else if $a_i \in \{B, V\}$

n_b += 1

else if $a_i \in \{D, H\}$

n_c += 1

else if $a_i = N$

n_d += 1

else if $a_i = S$

n_e += 1

else if $a_i = W$

n_f += 1

else if $a_i \in \{A, C, G, U\}$

n_g += 1

if $a_i = C$

num_C += 1

else //a_i must be G

num_G += 1

return $n_a, n_b, n_c, n_d, n_e, n_f, n_g, num_C, num_G$

def $computeNumberExternalLoops(n, k, \mathbf{a})$

//number external loops of size n with GC-content k given IUPAC constraints $\mathbf{a}$

$n_a, n_b, n_c, n_d, n_e, n_f, n_g, num_C, num_G = f(\mathbf{a})$

$k_0 = k - (num_C + num_G + n_e)$

$N = 0$

for $k_a = 0$ to n_a

for $k_b = 0$ to n_b

for $k_c = 0$ to n_c

$k_d = k_0 - (k_a + k_b + k_c)$

$C = \binom{n_a}{k_a} \cdot \binom{n_b}{k_b} \cdot \binom{n_c}{k_c} \cdot \binom{n_d}{k_d} \cdot 2^{n_c+n_d+n_e+n_f} \cdot 2^{k_b-k_c}$

N += C

return N
